# Supplementary material for: Analysis of Food Perception in Slim, Overweight, or Obese Individuals
Source: Nutrients. 2025 Jun 20;17(13):2054. doi: 10.3390/nu17132054 (PMC12250828; doi:10.3390/nu17132054)
Supplement: Supplementary file 1 [file nutrients-17-02054-s001.zip › nutrients-3683437-supplementary.pdf]

Questionnaire

We invite you to complete the following questionnaire
conducted as part of the study entitled „The influence of body mass index on the perception of the size of meal portions" carried out in the **Department od Diabetology and Internal Diseases at the University Clinical Center of the Medical University of Warsaw , Poland**, under the supervision of **Profesor Leszek Czupryniak, MD, PhD.**

The aim of the study is to determine the impact of BMI on
the size of consumed portions. The survey is anonymous. Please mark your honest answers. Participation in the survey is equal to consent to the processing of anonymized data for purposes related to this scientific research.

In the survey you will see 25 different meals. It takes a
maximum of 30 seconds to evaluate each of them. **Please rate each dish.**

* Wskazuje wymagane pytanie

1. Initials (first letter of name and surname) *

2. Body weight [kilograms] *

3. Growth [centimeters] *

4. Where are you from?

Zaznacz tylko jedną odpowiedź.

- Albania
- Austria
- Belgium
- Bulgaria
- Croatia
- Czech Republic
- Germany
- Israel
- Lithuania
- Netherlands
- North Macedonia
- Poland
- Portugal
- Romania
- Slovakia
- Serbia
- Slovenia
- Spain
- Switzerland
- United Kingdom
- Inne: ____________________

5. What kind of lifestyle do you have (do not include planned, additional physical activity such as gym, running, nordic-walking etc.)?

Zaznacz tylko jedną odpowiedź.

- at work and at home, the main guided lifestyle
- at work most of the day I stand/have a phisical work, later sedentary lifestyle
- at work I stand/have a phisical work, then I am still active (I take at least 7,000 steps)
- at work I mostly sit, but then I am active (I take at least 7,000 steps)

6. Do you have any additional, planned physical activity (e.g. running, gym, nordic-walking)?

Zaznacz tylko jedną odpowiedź.

- no
- yes, 1-2 times a week
- yes, 3-4 times a week
- yes, 5-7 times a week

7. **Sex** *

Zaznacz tylko jedną odpowiedź.

- Female
- Male

8. Age (years) *

____

9. Education *

Zaznacz tylko jedną odpowiedź.

- Elementary school
- Secondary school
- Technical
- Postsecondary education (bachelor/master/PhD degree etc.)

10. **Field of education** *

Zaznacz wszystkie właściwe odpowiedzi.

- medical sciences
- exact sciences
- electronics / IT
- economic sciences
- humanities
- agricultural sciences
- arts
- sport
- other

11. **Do you suffer from diabetes ?** *

Zaznacz tylko jedną odpowiedź.

- no
- I suffer from prediabetes
- Yes, diabetes mellitus type 1
- Yes, diabetes mellitus type 2
- Yes, diabetes mellitus typ 3 (e.g. diabetes after removal of the pancreas, diabetes after acute pancreatitis)

12. If you suffer from diabetes what was the **year** that it was diagnosed?

13. Are you pregnant or breastfeeding?

Zaznacz tylko jedną odpowiedź.

- no
- yes, first trimester
- yes, second trimester
- yes, third trimester
- breast-feeding

2 SEKCJA PYTAŃ

14. **My body weight according to BMI :** *

Zaznacz tylko jedną odpowiedź.

- is normal
- I'm underweight
- I'm overweight
- I'm obese
- I don't know

15. **How do you rate your diet ?** *

Zaznacz tylko jedną odpowiedź.

- I eat properly
- I eat incorrectly
- I do not care about this

16. How do you assess the size of the portions you consume? *

Zaznacz tylko jedną odpowiedź.

- I eat very little (1)
- I eat little (2)
- I eat according to my caloric needs (3)
- I eat a lot (4)
- I eat very large portions (5)

17. How many calories (estimated) do you consume per day? *

____

18. Do you know your daily calorie requirement? *

Zaznacz tylko jedną odpowiedź.

- Yes
- No

19. If you answered "yes" above, please provide your daily calorie requirement (in kcal)

____

20. Have you ever received dietary advice? *

Zaznacz tylko jedną odpowiedź.

- Yes
- No

21. **Have you ever been on reduction diet (slimming diet) ?** *

Zaznacz tylko jedną odpowiedź.

- Yes
- No

22. If you answered "yes" above, how long (maximum) have you been on a reducing diet?

Zaznacz tylko jedną odpowiedź.

- A few days, less than a week
- From 1 week, less than 2 weeks
- 2-3 weeks
- Over 3 weeks to a month
- Longer than a month

23. How many meals do you eat per day (main meals plus snacks)? *

Zaznacz tylko jedną odpowiedź.

- From 1 to 2 meals
- From 3 to 4 meals
- From 5 to 6 meals
- I don't count meals

24. When preparing the same meals, do you always eat similar portions of products? *

Zaznacz tylko jedną odpowiedź.

- Yes
- No

25. Do you control the size of the portions of food you eat? *

Zaznacz tylko jedną odpowiedź.

- ( ) Yes
- ( ) No, I eat as much food as I want

26. If you control the portion of meals you eat, why do you do it (multiple answers possible):

Zaznacz wszystkie właściwe odpowiedzi.

- [ ] I want to reduce my body weight
- [ ] I don't want to overeat
- [ ] Inne: ____________

27. When do you assess the size of the consumed portion of meal , what do you use (multiple answers possible): *

Zaznacz wszystkie właściwe odpowiedzi.

- [ ] visual assessment of portions
- [ ] I count calories
- [ ] I weigh the products
- [ ] I use the recommended portions for consumption from the food label of the products
- [ ] I prepare meals according to a diet designed individually for me
- [ ] Inne: ____________

28. When you feel more hungry before a meal, do you increase the volume of your meal? *

Zaznacz tylko jedną odpowiedź.

- ( ) Yes
- ( ) No

29. If you eat out/take food away, you usually eat: *

Zaznacz tylko jedną odpowiedź.

- Whole
- Not the whole meal

30. If you answered "not the whole meal" above, why don't you usually eat the whole meal?

Zaznacz tylko jedną odpowiedź.

- I consider the portions of the dish to be too large for my needs, even though I DON'T WANT to lose weight
- I consider the portions of the dish to be too large for my needs, and I WANT to lose weight
- I get full before the entire meal is finished, so I stop eating
- the meal is tasteless
- Inne: ______________________

31. How do you rate your health? *

Zaznacz tylko jedną odpowiedź.

Bad

1
2
3
4
5
6
7
8
9
10

Very good

32. When did you eat your last meal?

Zaznacz tylko jedną odpowiedź.

- in the last 30 minutes
- from 30 min. up to 1 hour
- between 1 and 2 hours
- between 2 and 3 hours
- over 3 hours ago

33. Do you use medications that affect your appetite? (e.g. Mysimba, Victoza, Saxenda, Trulicity, Ozempic, Rybelsus) *

Zaznacz tylko jedną odpowiedź.

- Yes
- No

34. If you use medications that affect your appetite, please enter the name of the medication

---

OCENA WYBRANYCH POSIŁKÓW 1/3

In the survey you will see 25 different meals. Each assessment takes a maximum of 30 seconds. Please rate each dish.

The products are always on the same plate and the photos are taken from the same perspective. There is cutlery nearby to help you estimate portions.

Hamburger with fries (pork, melted cheese, ketchup, mustard, onion, pickles)

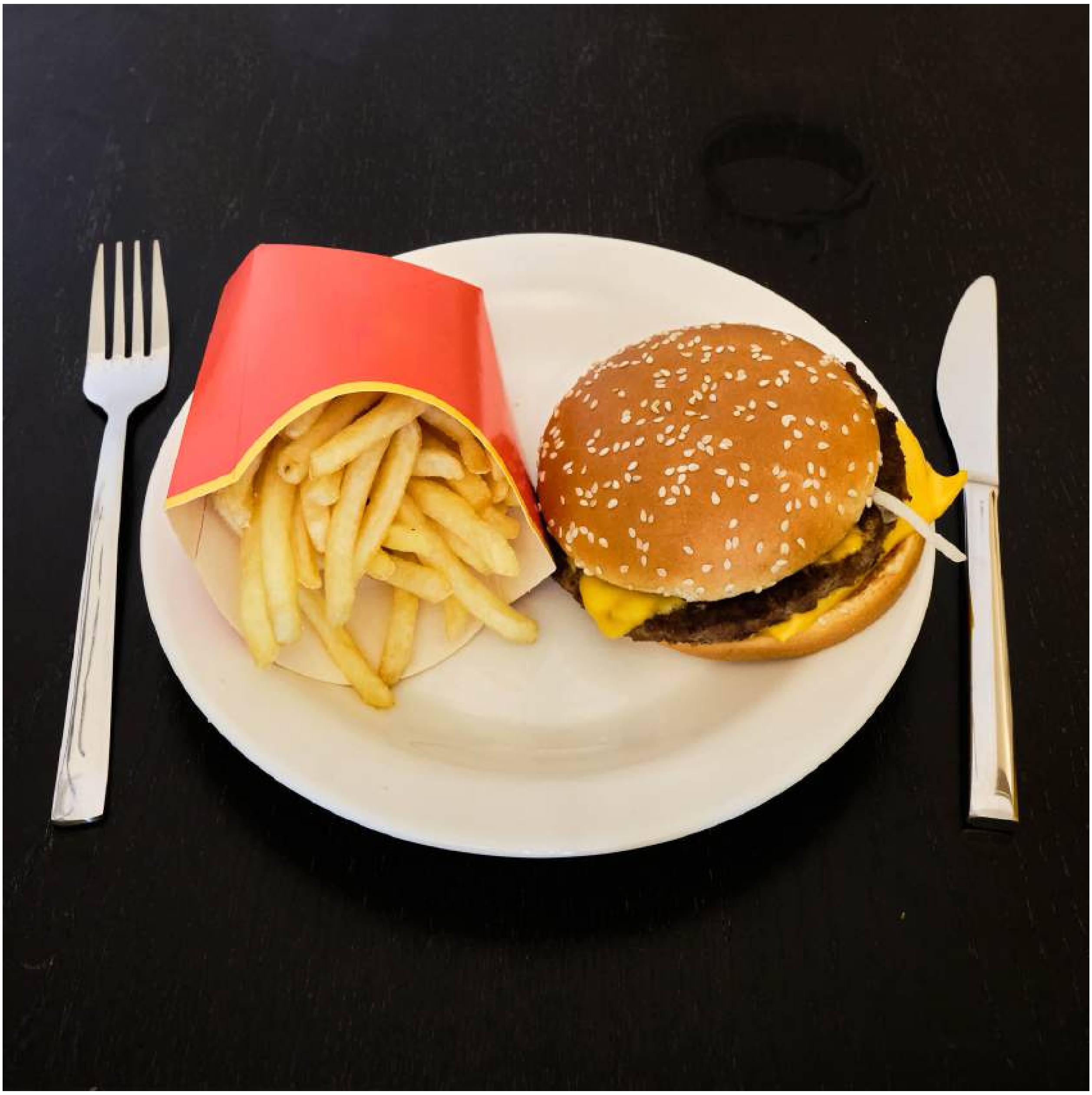

35. Determine the size of the meal shown in the photo *

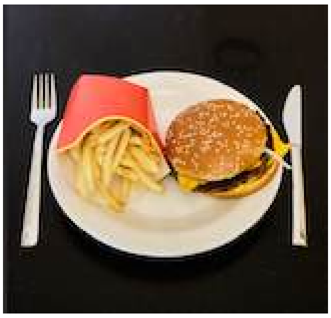

Zaznacz tylko jedną odpowiedź.

	Very small, almost nothing
1	◯
2	◯
3	◯
4	◯
5	◯
6	◯
7	◯
8	◯
9	◯
10	◯
	Very Large

36. How full would you be with this meal? *

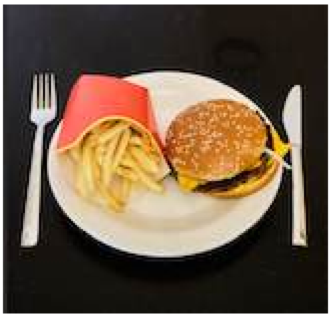

Zaznacz tylko jedną odpowiedź.

Not full at all, still hungry

1 ○
2 ○
3 ○
4 ○
5 ○
6 ○
7 ○
8 ○
9 ○
10 ○

Extremely full, on the verge of overeating

37. Do you think the meal in the photo is healthy? *

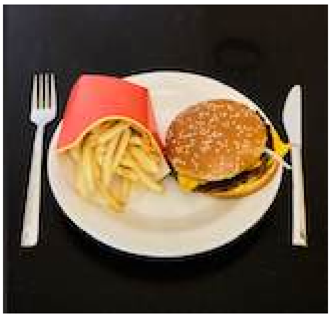

Zaznacz tylko jedną odpowiedź.

- Yes
- No

38. Would you still be hungry immediately after eating this meal? *

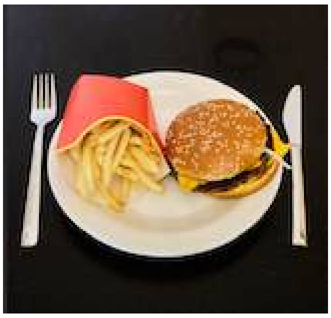

Zaznacz tylko jedną odpowiedź.

- Yes
- No

39. Do you think this meal is for you: *

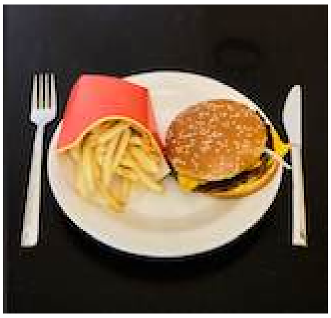

Zaznacz tylko jedną odpowiedź.

- too caloric
- has the right amount of kcal
- it has too few calories, I can or should eat a larger portion

40. How many calories do you think the meal in the photo has? *

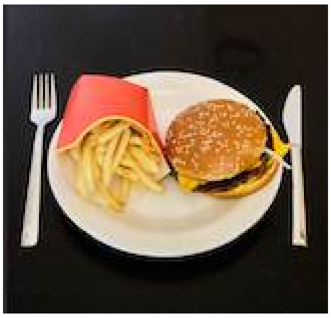

41. How long after eating a meal would you start feeling hungry again? *

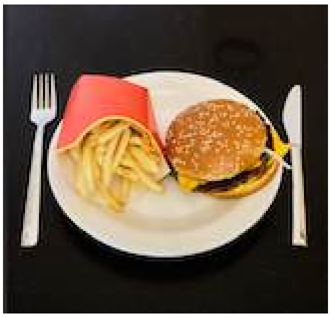

Zaznacz tylko jedną odpowiedź.

- Before the hour is up
- After 1 hour to 2 hours
- Over 2 hours, up to 3 hours
- Over 3 hours

Croissant with black coffee without sugar

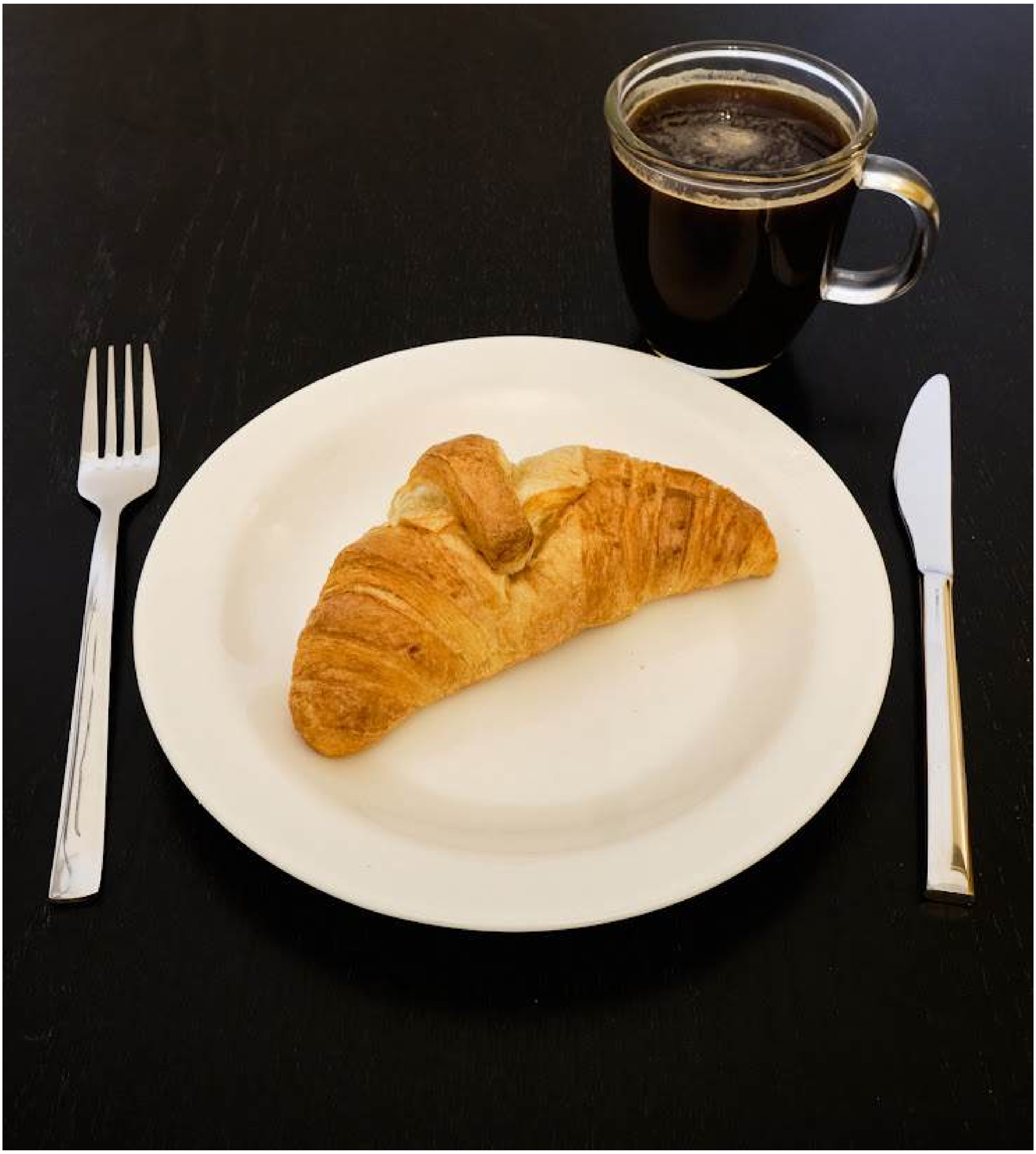

42. Determine the size of the meal shown in the photo *

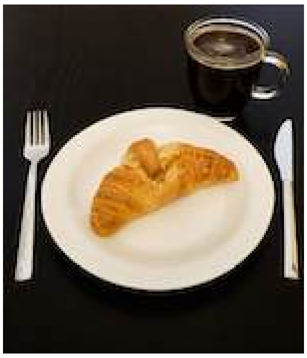

Zaznacz tylko jedną odpowiedź.

	Very small, almost nothing
1	
2	
3	
4	
5	
6	
7	
8	
9	
10	
	Very Large

43. How full would you be with this meal? *

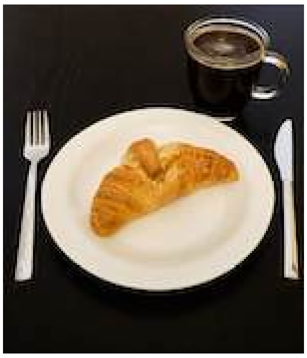

Zaznacz tylko jedną odpowiedź.

Not full at all, still hungry
1 ☐
2 ☐
3 ☐
4 ☐
5 ☐
6 ☐
7 ☐
8 ☐
9 ☐
10 ☐
Extremely full, on the verge of overeating

44. Do you think the meal in the photo is healthy? *

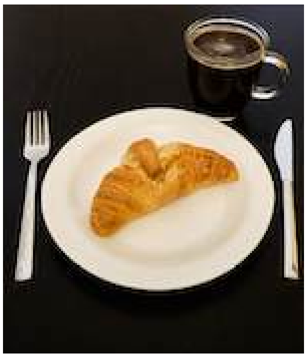

Zaznacz tylko jedną odpowiedź.

- Yes
- No

45. Would you still be hungry immediately after eating this meal? *

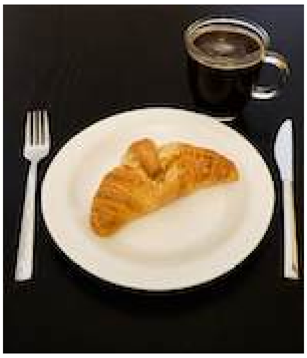

Zaznacz tylko jedną odpowiedź.

- Yes
- No

46. Do you think this meal is for you: *

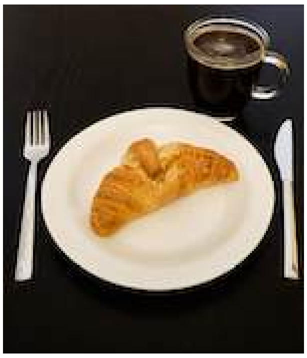

Zaznacz tylko jedną odpowiedź.

- too caloric
- has the right amount of kcal
- it has too few calories, I can or should eat a larger portion

47. How many calories do you think the meal in the photo has? *

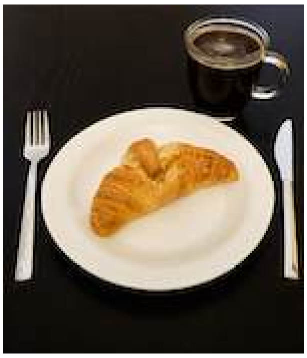

48. How long after eating the meal would you start feeling hungry again? *

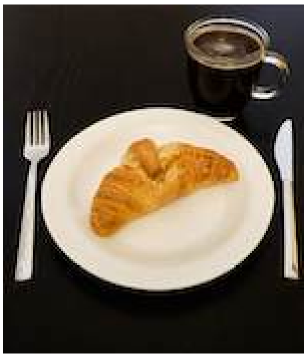

Zaznacz tylko jedną odpowiedź.

- Before the hour is up
- After 1 hour to 2 hours
- Over 2 hours, up to 3 hours
- Over 3 hours

Sandwiches made of whole grain bread (wheat and rye) with low-fat cottage cheese, pumpkin seeds, peppers and tomatoes

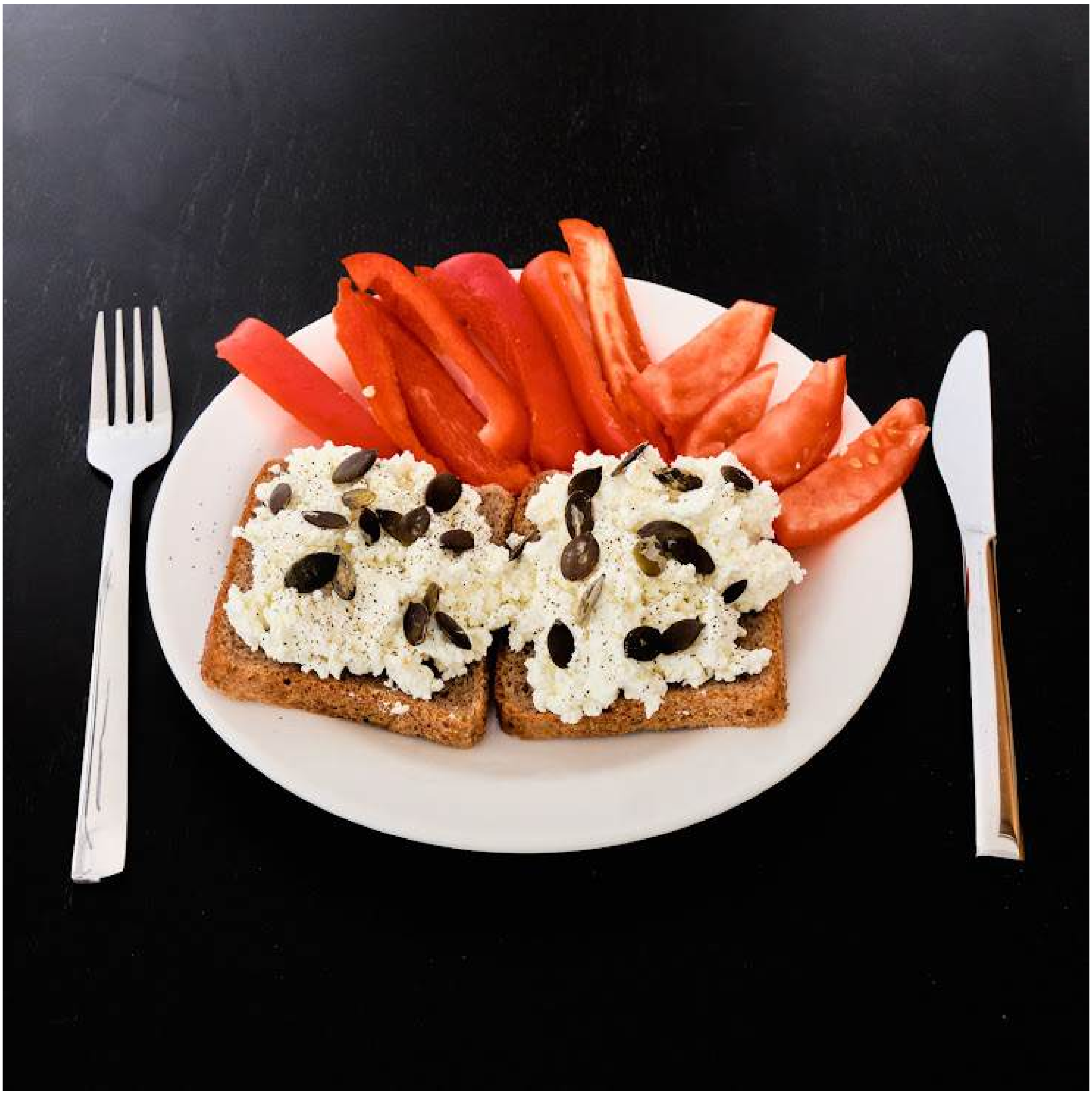

49. Determine the size of the meal shown in the photo *

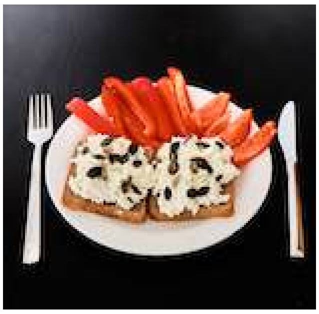

Zaznacz tylko jedną odpowiedź.

	Very small, almost nothing
1	◯
2	◯
3	◯
4	◯
5	◯
6	◯
7	◯
8	◯
9	◯
10	◯
	Very Large

50. How full would you be with this meal? *

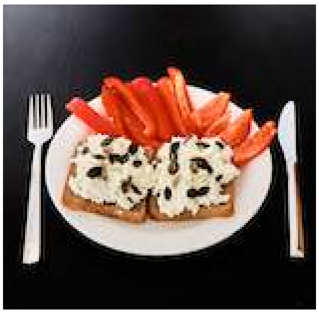

Zaznacz tylko jedną odpowiedź.

Not full at all, still hungry

1

2

3

4

5

6

7

8

9

10

Extremely full, on the verge of overeating

51. Do you think the meal in the photo is healthy? *

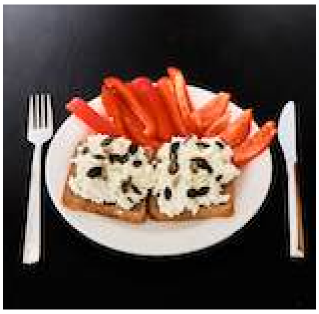

Zaznacz tylko jedną odpowiedź.

- Yes
- No

52. Would you still be hungry immediately after eating this meal? *

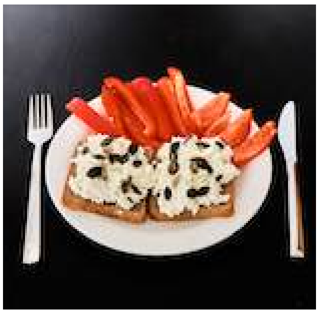

Zaznacz tylko jedną odpowiedź.

- Yes
- No

53. Do you think this meal is for you: *

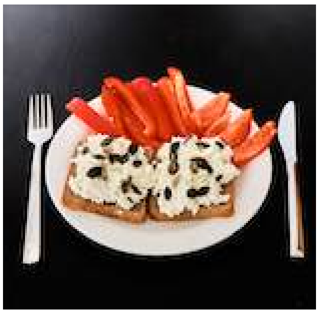

Zaznacz tylko jedną odpowiedź.

- too caloric
- has the right amount of kcal
- it has too few calories, I can or should eat a larger portion

54. How many calories do you think the meal in the photo has? *

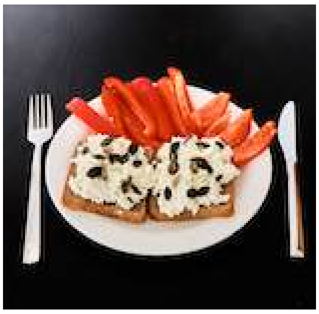

55. How long after eating the meal would you start feeling hungry again? *

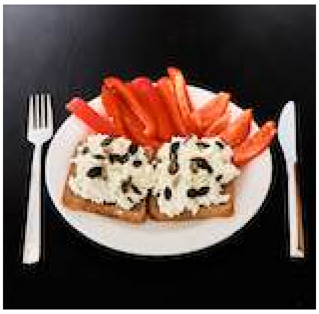

Zaznacz tylko jedną odpowiedź.

- Before the hour is up
- After 1 hour to 2 hours
- Over 2 hours, up to 3 hours
- Over 3 hours

Sushi

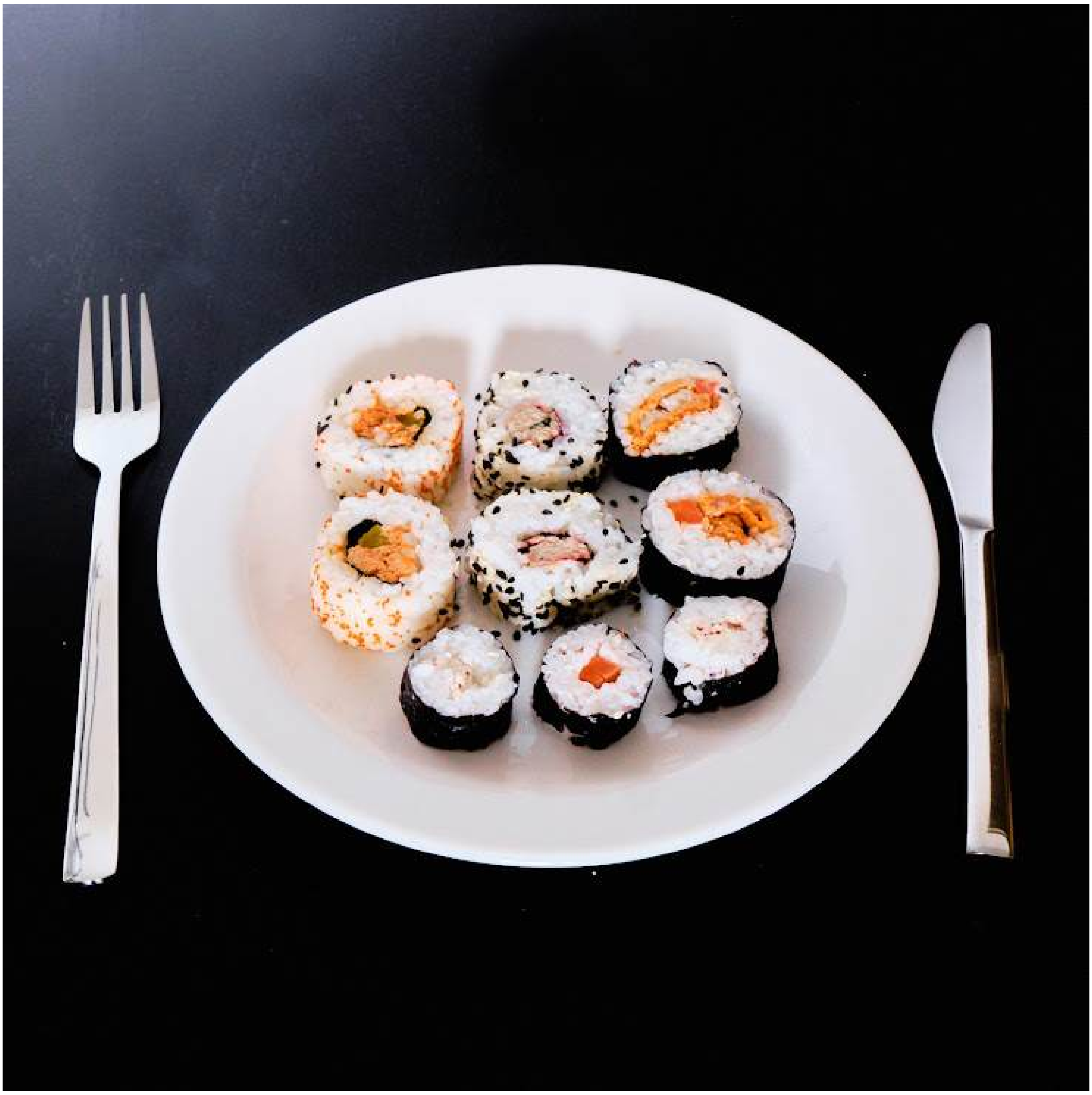

56. Determine the size of the meal shown in the photo *

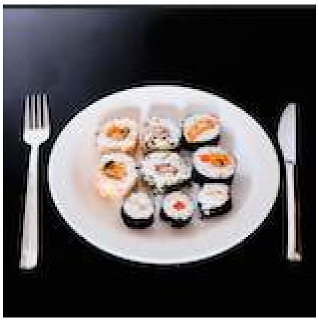

Zaznacz tylko jedną odpowiedź.

	Very small, almost nothing
1	⬭
2	⬭
3	⬭
4	⬭
5	⬭
6	⬭
7	⬭
8	⬭
9	⬭
10	⬭
	Very Large

57. How full would you be with this meal? *

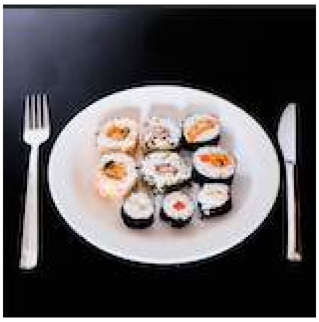

Zaznacz tylko jedną odpowiedź.

Not full at all, still hungry	
1	
2	
3	
4	
5	
6	
7	
8	
9	
10	

Extremely full, on the verge of overeating

58. Do you think the meal in the photo is healthy? *

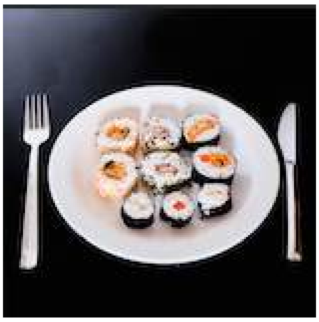

Zaznacz tylko jedną odpowiedź.

- Yes
- No

59. Would you still be hungry immediately after eating this meal? *

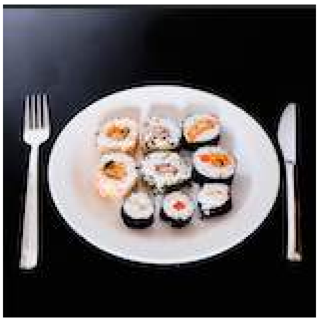

Zaznacz tylko jedną odpowiedź.

- Yes
- No

60. Do you think this meal is for you: *

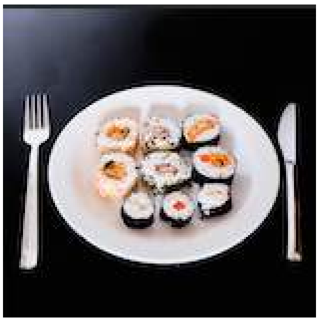

Zaznacz tylko jedną odpowiedź.

- too caloric
- has the right amount of kcal
- it has too few calories, I can or should eat a larger portion

61. How many calories do you think the meal in the photo has? *

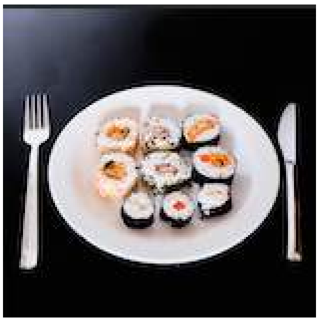

____

62. How long after eating the meal would you start feeling hungry again? *

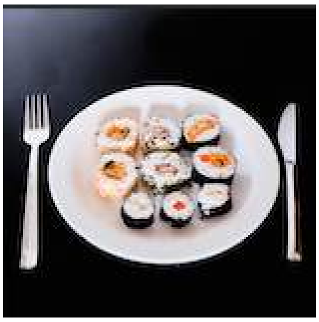

Zaznacz tylko jedną odpowiedź.

- Before the hour is up
- After 1 hour to 2 hours
- Over 2 hours, up to 3 hours
- Over 3 hours

Minced cutlet (pork meat) in a spoon of rapeseed oil with boiled potatoes and cucumber salad (with 12% cream)

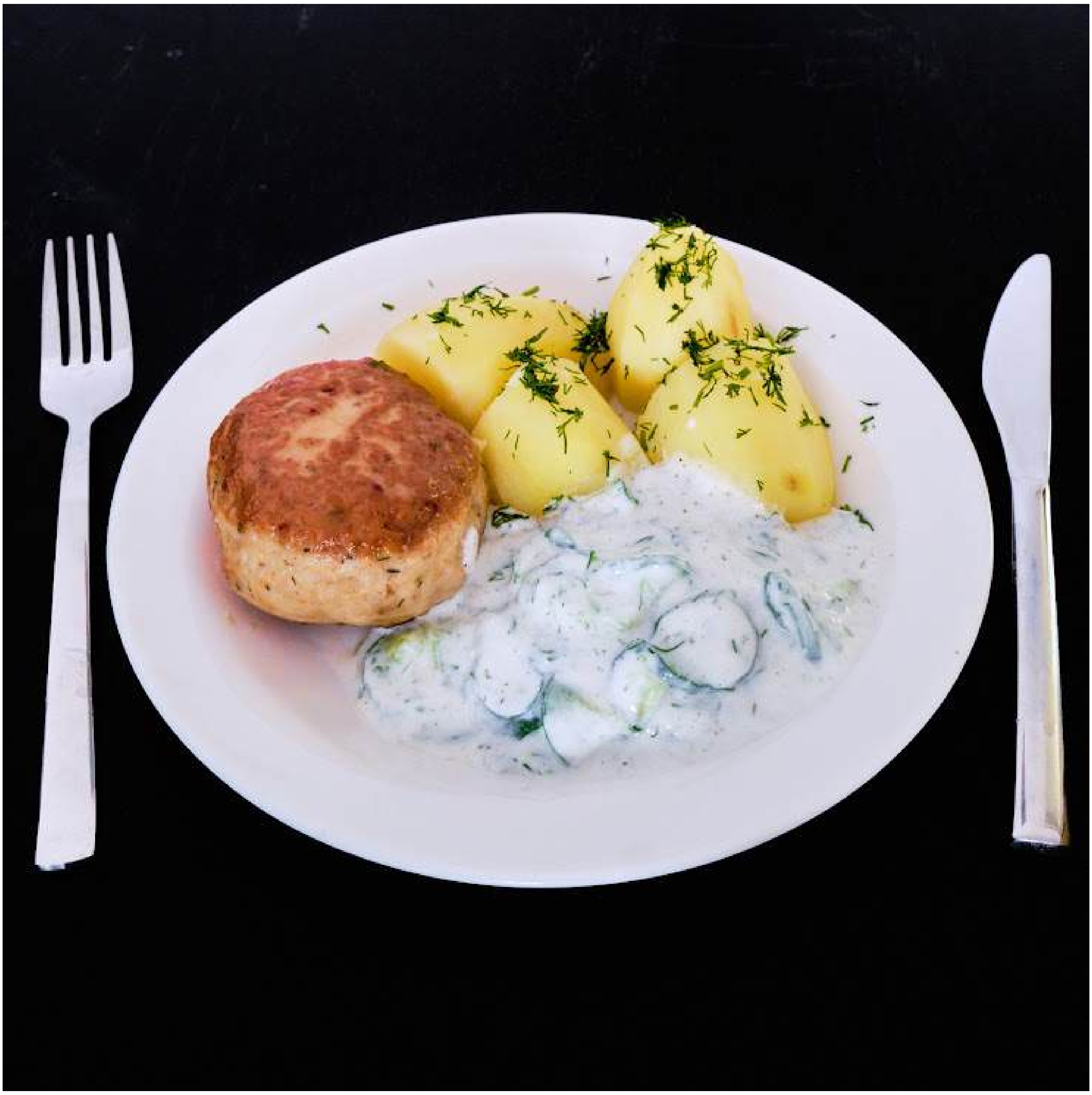

63. Determine the size of the meal shown in the photo *

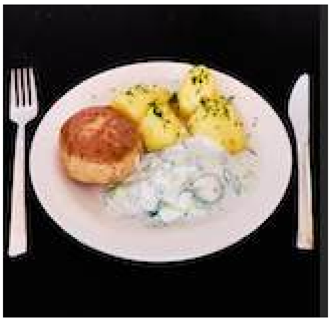

Zaznacz tylko jedną odpowiedź.

	Very small, almost nothing
1	◯
2	◯
3	◯
4	◯
5	◯
6	◯
7	◯
8	◯
9	◯
10	◯
	Very Large

64. How full would you be with this meal? *

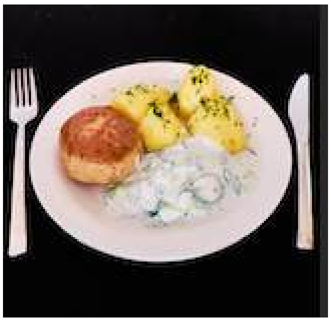

Zaznacz tylko jedną odpowiedź.

	Not full at all, still hungry
1	◯
2	◯
3	◯
4	◯
5	◯
6	◯
7	◯
8	◯
9	◯
10	◯
	Extremely full, on the verge of overeating

65. Do you think the meal in the photo is healthy? *

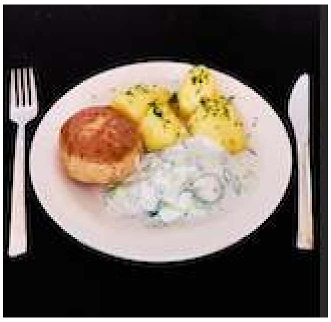

Zaznacz tylko jedną odpowiedź.

- Yes
- No

66. Would you still be hungry immediately after eating this meal? *

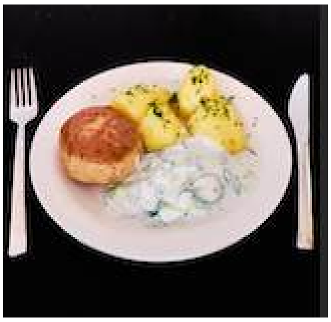

Zaznacz tylko jedną odpowiedź.

- Yes
- No

67. Do you think this meal is for you: *

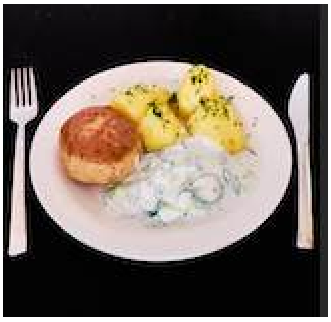

Zaznacz tylko jedną odpowiedź.

- too caloric
- has the right amount of kcal
- it has too few calories, I can or should eat a larger portion

68. How many calories do you think the meal in the photo has? *

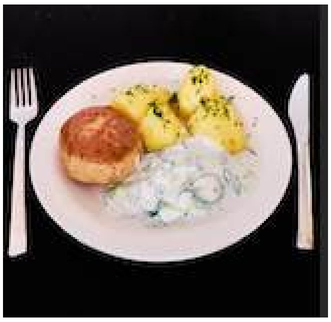

69. How long after eating the meal would you start feeling hungry again? *

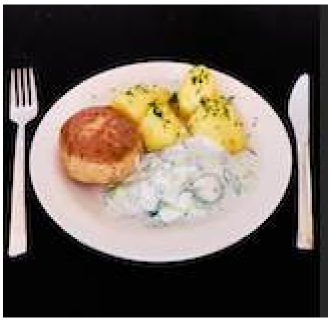

Zaznacz tylko jedną odpowiedź.

- Before the hour is up
- After 1 hour to 2 hours
- Over 2 hours, up to 3 hours
- Over 3 hours

Vodka

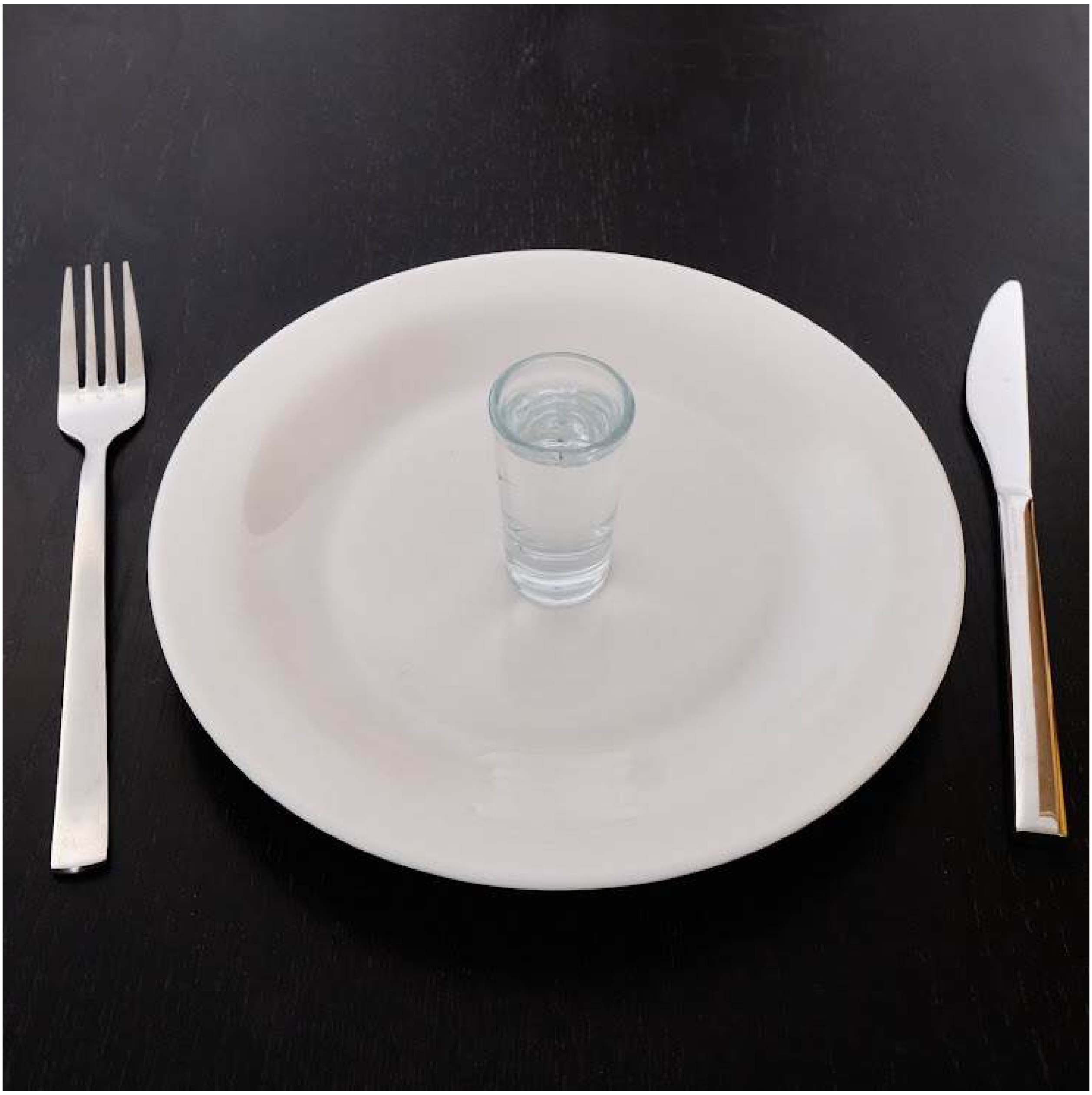

70. Determine the size of the meal shown in the photo *

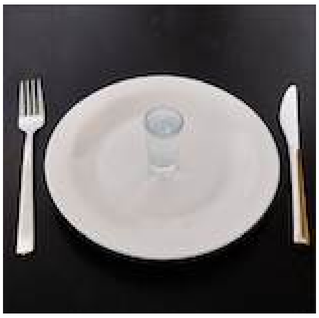

Zaznacz tylko jedną odpowiedź.

Very small, almost nothing

1 ⬭

2 ⬭

3 ⬭

4 ⬭

5 ⬭

6 ⬭

7 ⬭

8 ⬭

9 ⬭

10 ⬭

Very Large

71. How full would you be with this meal? *

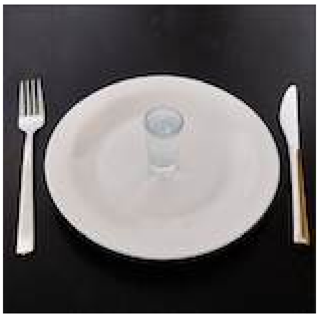

Zaznacz tylko jedną odpowiedź.

	Not full at all, still hungry
1	☐
2	☐
3	☐
4	☐
5	☐
6	☐
7	☐
8	☐
9	☐
10	☐
	Extremely full, on the verge of overeating

72. Do you think the meal in the photo is healthy? *

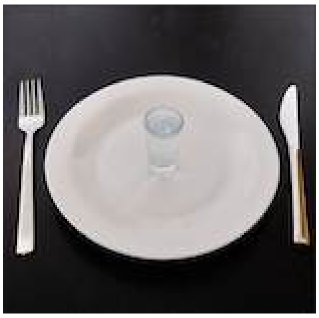

Zaznacz tylko jedną odpowiedź.

- Yes
- No

73. Would you still be hungry immediately after eating this meal? *

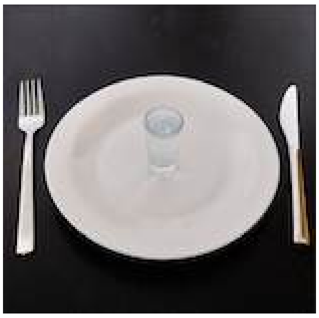

Zaznacz tylko jedną odpowiedź.

- Yes
- No

74. Do you think this meal is for you: *

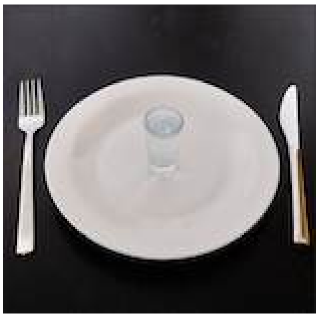

Zaznacz tylko jedną odpowiedź.

- too caloric
- has the right amount of kcal
- it has too few calories, I can or should eat a larger portion

75. How many calories do you think the meal in the photo has? *

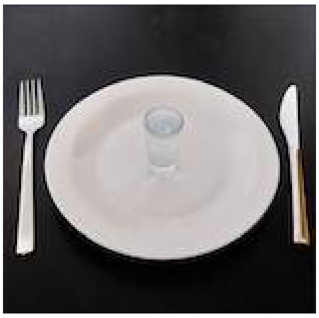

76. How long after eating the meal would you start feeling hungry again? *

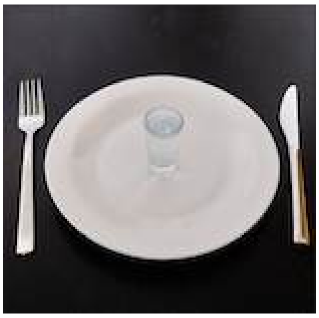

Zaznacz tylko jedną odpowiedź.

- Before the hour is up
- After 1 hour to 2 hours
- Over 2 hours, up to 3 hours
- Over 3 hours

Greek salad (with feta cheese, vegetables, a spoon of olive oil)

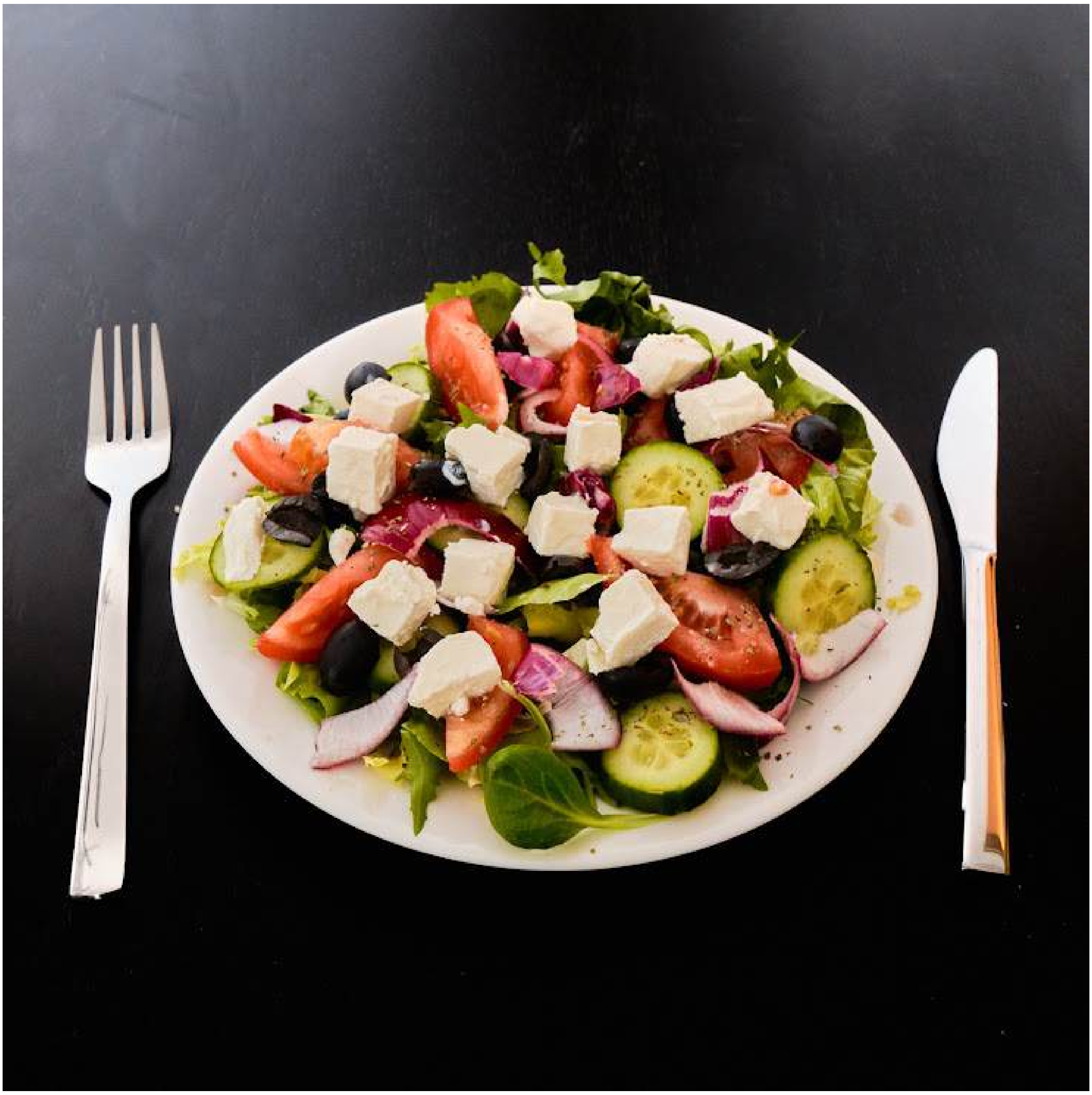

77. Determine the size of the meal shown in the photo *

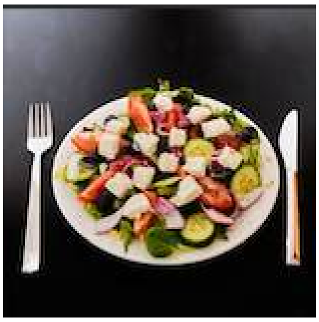

Zaznacz tylko jedną odpowiedź.

	Very small, almost nothing
1	⬭
2	⬭
3	⬭
4	⬭
5	⬭
6	⬭
7	⬭
8	⬭
9	⬭
10	⬭
	Very Large

78. How full would you be with this meal? *

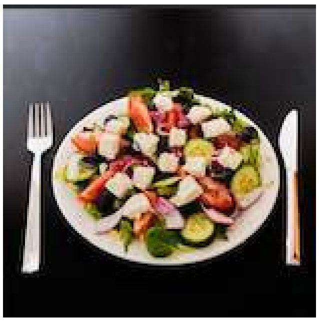

Zaznacz tylko jedną odpowiedź.

	Not full at all, still hungry
1	◯
2	◯
3	◯
4	◯
5	◯
6	◯
7	◯
8	◯
9	◯
10	◯
	Extremely full, on the verge of overeating

79. Do you think the meal in the photo is healthy? *

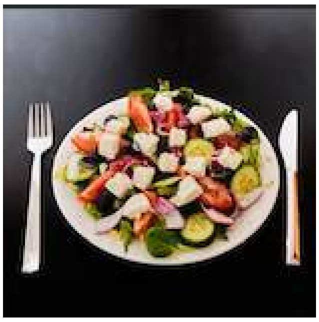

Zaznacz tylko jedną odpowiedź.

- Yes
- No

80. Would you still be hungry immediately after eating this meal? *

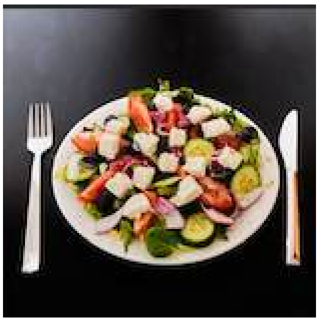

Zaznacz tylko jedną odpowiedź.

- Yes
- No

81. Do you think this meal is for you: *

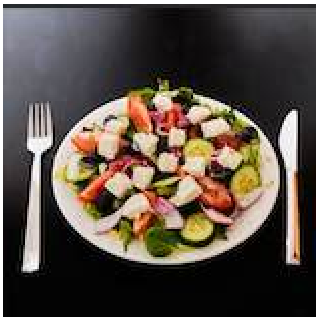

Zaznacz tylko jedną odpowiedź.

- too caloric
- has the right amount of kcal
- it has too few calories, I can or should eat a larger portion

82. How many calories do you think the meal in the photo has? *

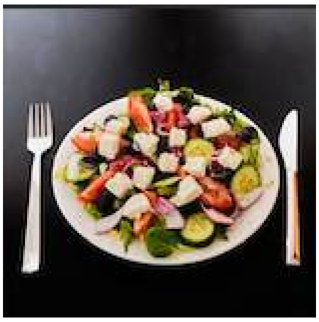

83. How long after eating the meal would you start feeling hungry again? *

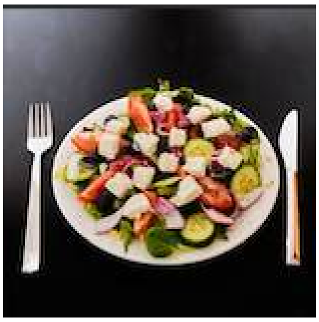

Zaznacz tylko jedną odpowiedź.

- Before the hour is up
- After 1 hour to 2 hours
- Over 2 hours, up to 3 hours
- Over 3 hours

Cheesecake

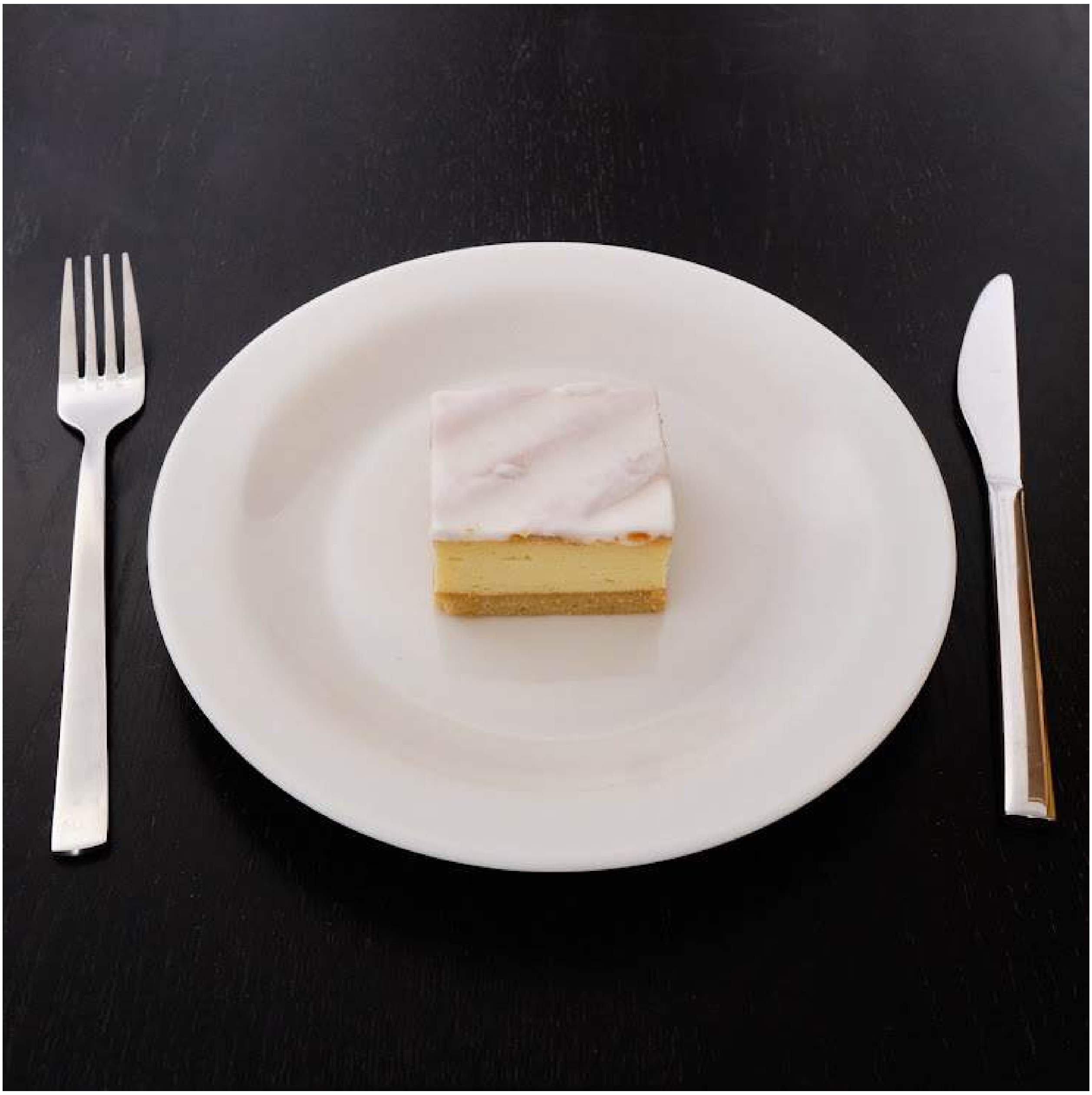

84. Determine the size of the meal shown in the photo *

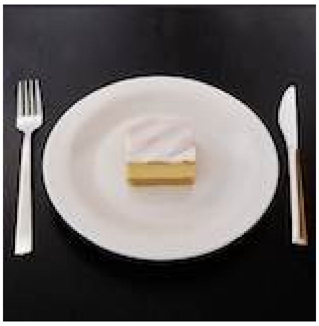

Zaznacz tylko jedną odpowiedź.

Very small, almost nothing

1
2
3
4
5
6
7
8
9
10

Very Large

85. How full would you be with this meal? *

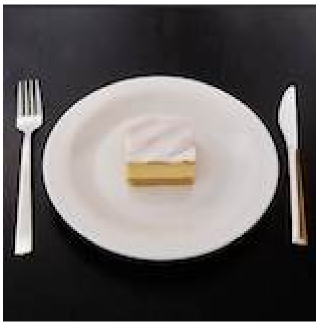

Zaznacz tylko jedną odpowiedź.

Not full at all, still hungry

1

2

3

4

5

6

7

8

9

10

Extremely full, on the verge of overeating

86. Do you think the meal in the photo is healthy? *

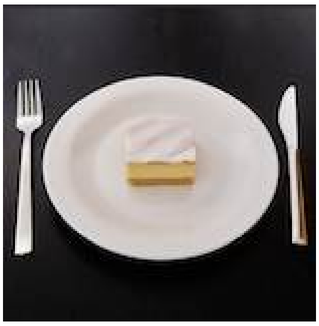

Zaznacz tylko jedną odpowiedź.

- Yes
- No

87. Would you still be hungry immediately after eating this meal? *

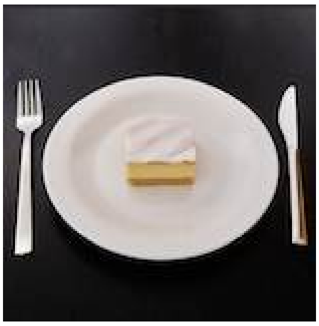

Zaznacz tylko jedną odpowiedź.

- Yes
- No

88. Do you think this meal is for you: *

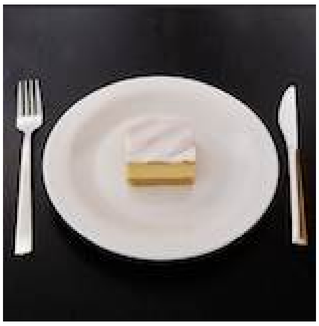

Zaznacz tylko jedną odpowiedź.

- too caloric
- has the right amount of kcal
- it has too few calories, I can or should eat a larger portion

89. How many calories do you think the meal in the photo has? *

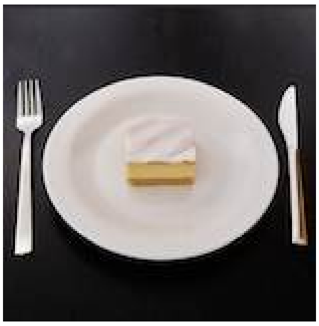

____

90. How long after eating the meal would you start feeling hungry again? *

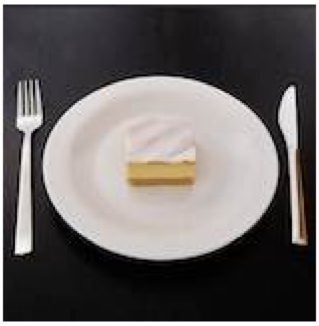

Zaznacz tylko jedną odpowiedź.

- Before the hour is up
- After 1 hour to 2 hours
- Over 2 hours, up to 3 hours
- Over 3 hours

Pizza with salami and pepperoni

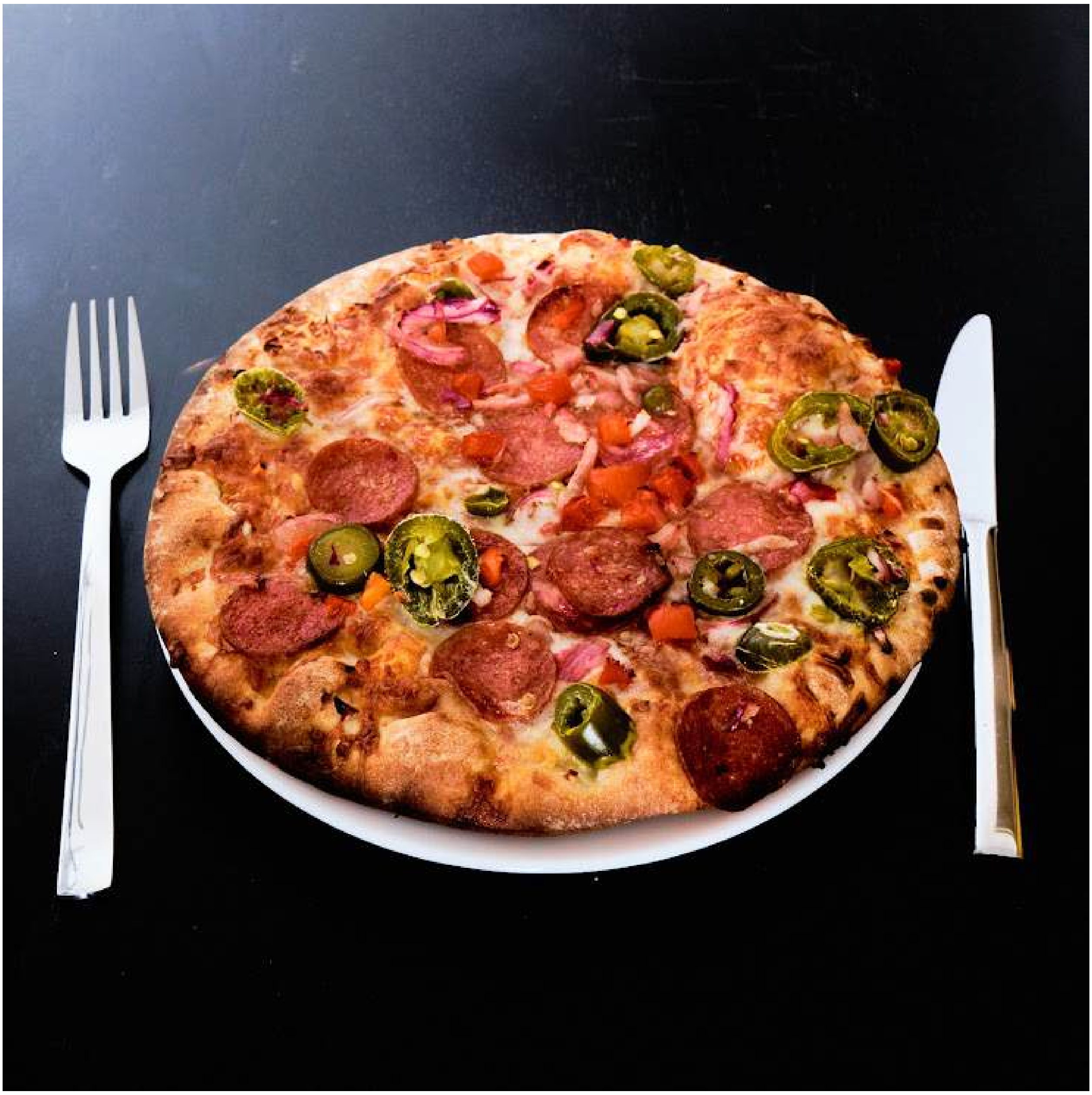

91. Determine the size of the meal shown in the photo *

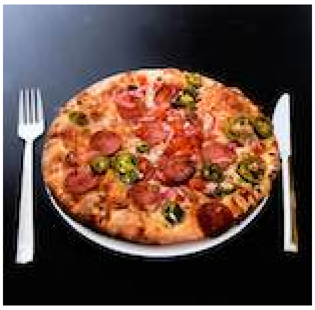

Zaznacz tylko jedną odpowiedź.

	Very small, almost nothing
1	⬭
2	⬭
3	⬭
4	⬭
5	⬭
6	⬭
7	⬭
8	⬭
9	⬭
10	⬭
	Very Large

92. How full would you be with this meal? *

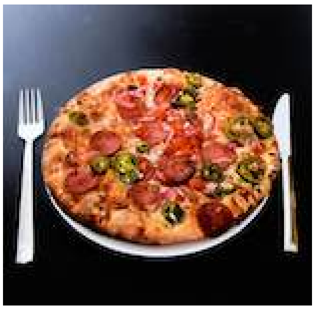

Zaznacz tylko jedną odpowiedź.

Not full at all, still hungry

1 ◯

2 ◯

3 ◯

4 ◯

5 ◯

6 ◯

7 ◯

8 ◯

9 ◯

10 ◯

Extremely full, on the verge of overeating

93. Do you think the meal in the photo is healthy? *

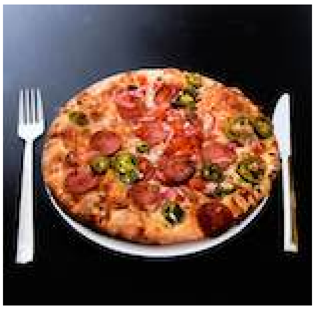

Zaznacz tylko jedną odpowiedź.

- Yes
- No

94. Would you still be hungry immediately after eating this meal? *

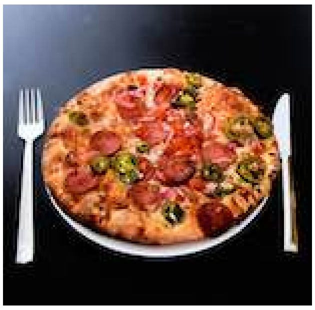

Zaznacz tylko jedną odpowiedź.

- Yes
- No

95. Do you think this meal is for you: *

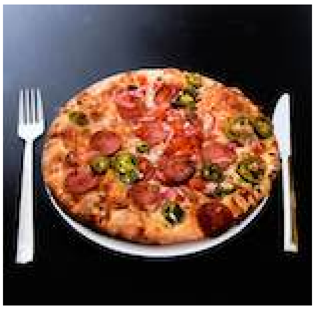

Zaznacz tylko jedną odpowiedź.

- too caloric
- has the right amount of kcal
- it has too few calories, I can or should eat a larger portion

96. How many calories do you think the meal in the photo has? *

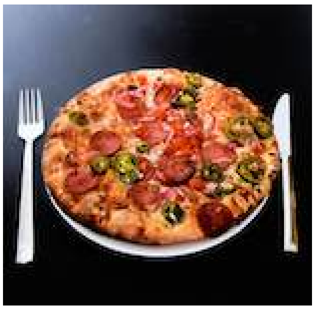

97. How long after eating the meal would you start feeling hungry again? *

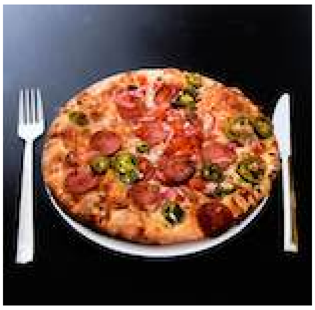

Zaznacz tylko jedną odpowiedź.

- Before the hour is up
- After 1 hour to 2 hours
- Over 2 hours, up to 3 hours
- Over 3 hours

OCENA WYBRANYCH POSIŁKÓW 2/3

The products are always on the same plate and the photos are taken from the same perspective. There is cutlery nearby to help you estimate portions.

A handful of raisins

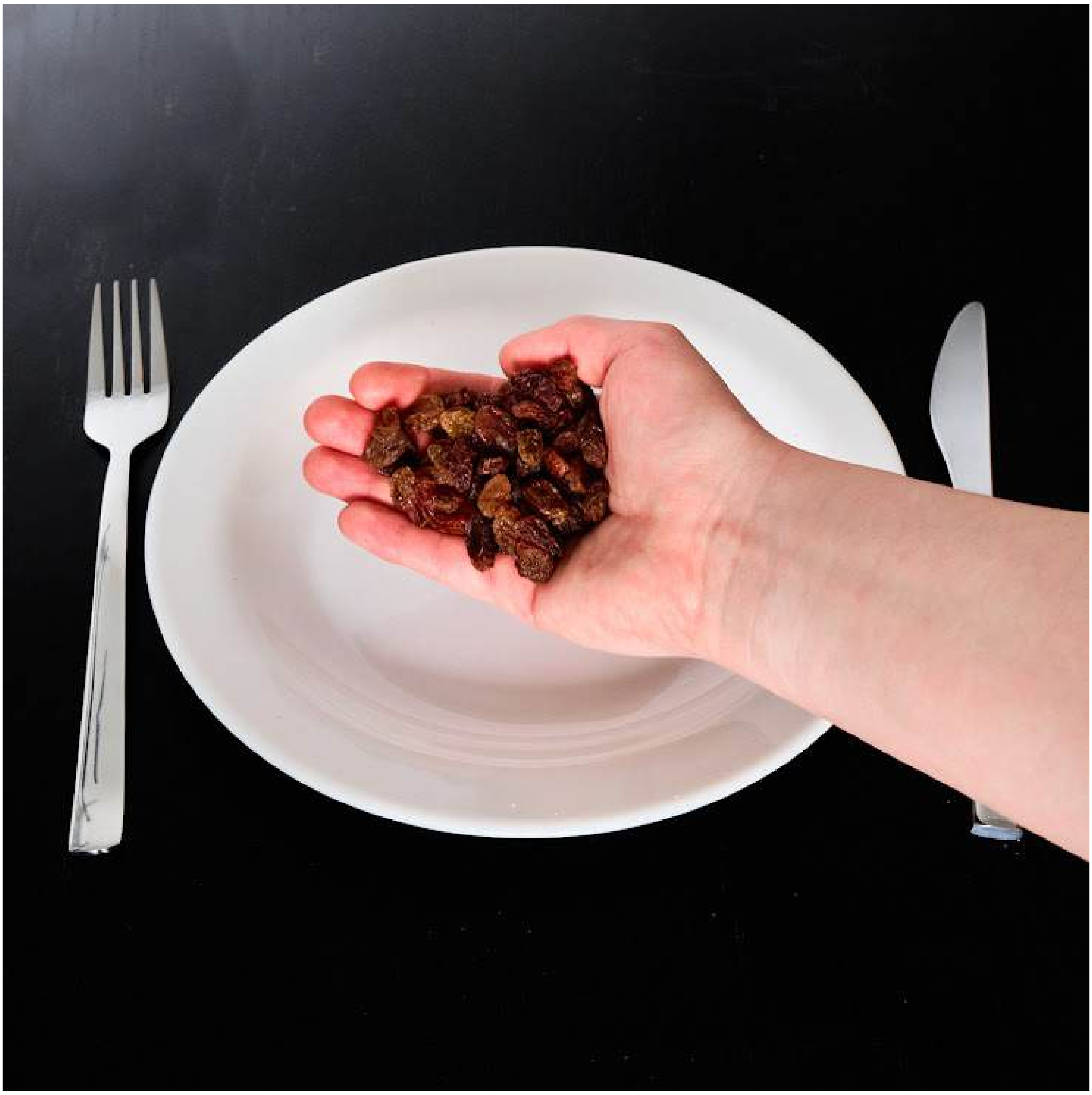

98. Determine the size of the meal shown in the photo *

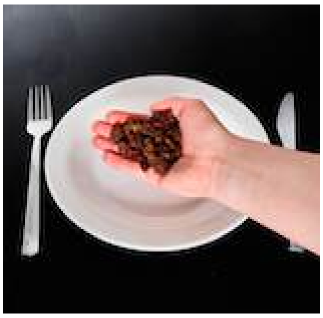

Zaznacz tylko jedną odpowiedź.

Very small, almost nothing	
1	⬭
2	⬭
3	⬭
4	⬭
5	⬭
6	⬭
7	⬭
8	⬭
9	⬭
10	⬭
Very Large	

99. How full would you be with this meal? *

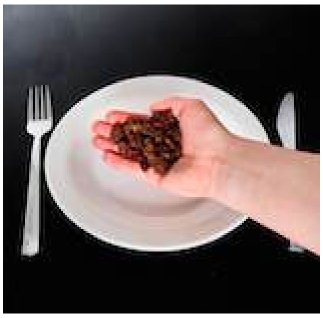

Zaznacz tylko jedną odpowiedź.

Not full at all, still hungry
1
2
3
4
5
6
7
8
9
10
Extremely full, on the verge of overeating

100. Do you think the meal in the photo is healthy? *

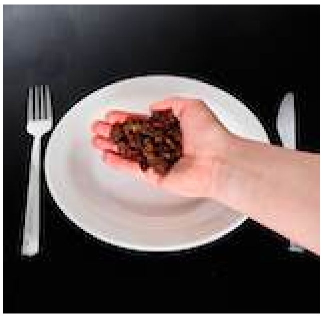

Zaznacz tylko jedną odpowiedź.

- Yes
- No

101. Would you still be hungry immediately after eating this meal? *

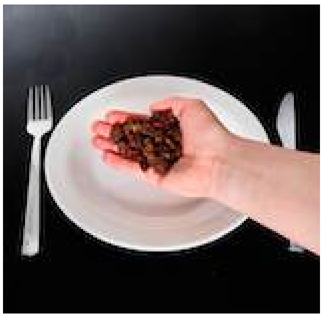

Zaznacz tylko jedną odpowiedź.

- Yes
- No

102. Do you think this meal is for you: *

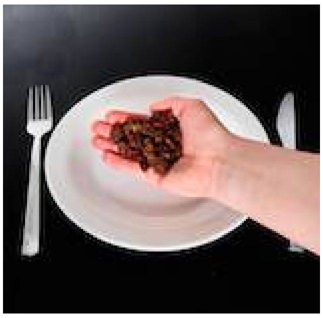

Zaznacz tylko jedną odpowiedź.

- too caloric
- has the right amount of kcal
- it has too few calories, I can or should eat a larger portion

103. How many calories do you think the meal in the photo has? *

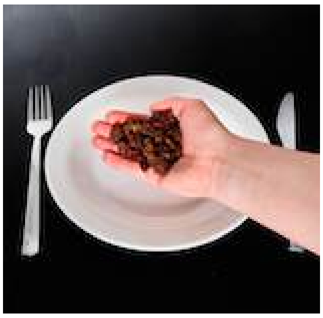

104. How long after eating the meal would you start feeling hungry again? *

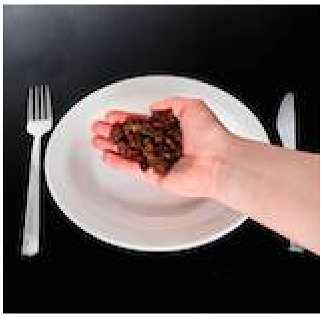

Zaznacz tylko jedną odpowiedź.

- Before the hour is up
- After 1 hour to 2 hours
- Over 2 hours, up to 3 hours
- Over 3 hours

Red wine

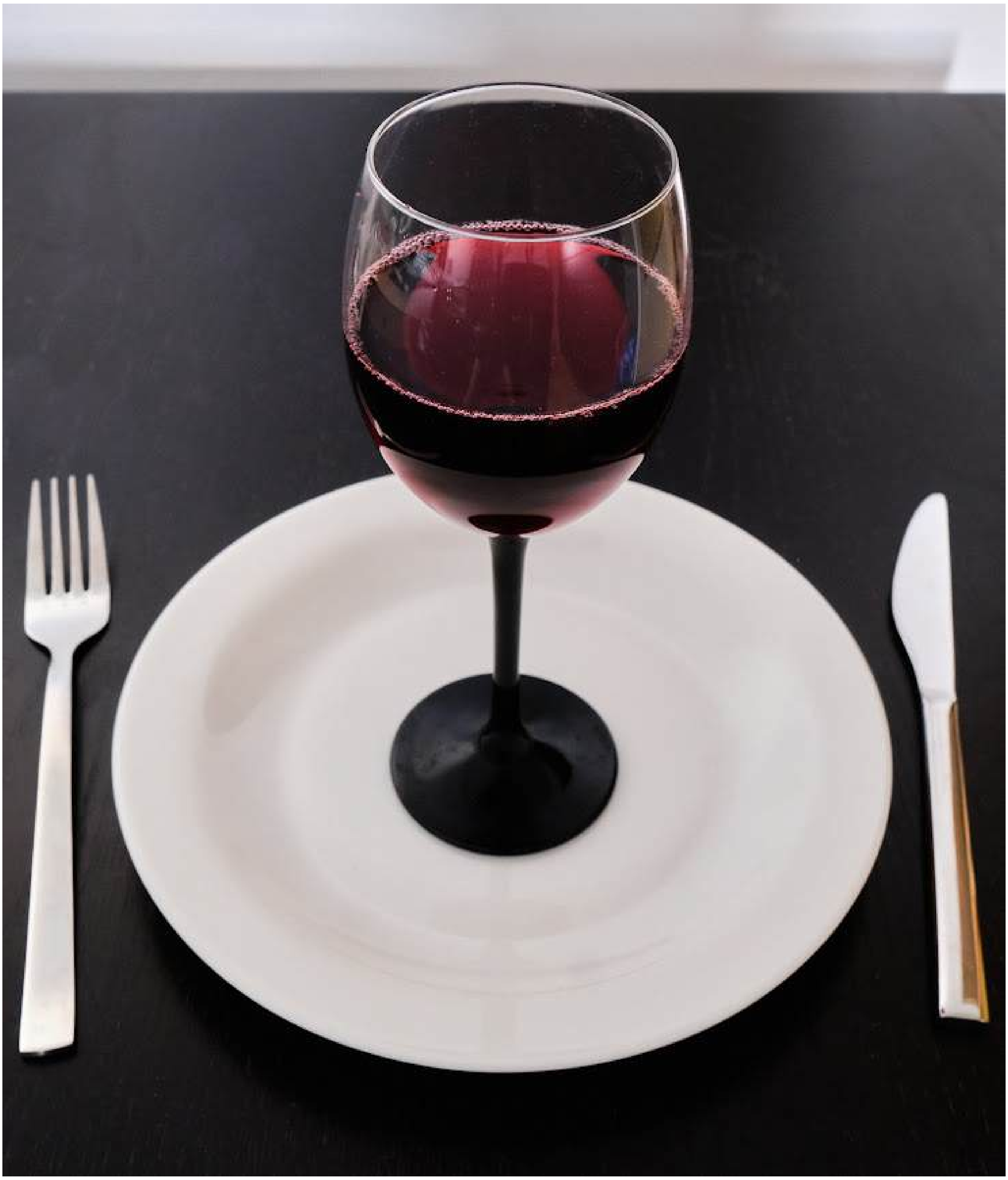

105. Determine the size of the meal shown in the photo *

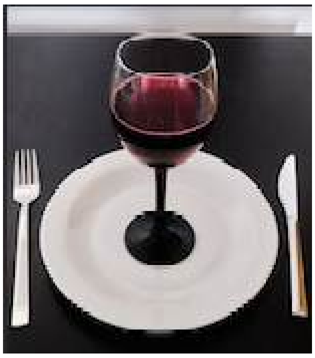

Zaznacz tylko jedną odpowiedź.

Very small, almost nothing

1

2

3

4

5

6

7

8

9

10

Very Large

106. How full would you be with this meal? *

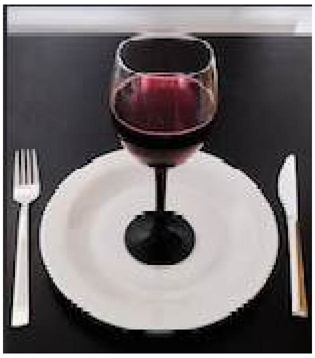

Zaznacz tylko jedną odpowiedź.

	Not full at all, still hungry
1	◯
2	◯
3	◯
4	◯
5	◯
6	◯
7	◯
8	◯
9	◯
10	◯
	Extremely full, on the verge of overeating

107. Do you think the meal in the photo is healthy? *

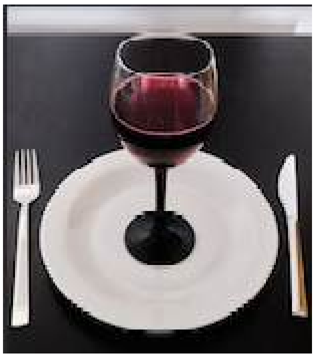

Zaznacz tylko jedną odpowiedź.

- Yes
- No

108. Would you still be hungry immediately after eating this meal? *

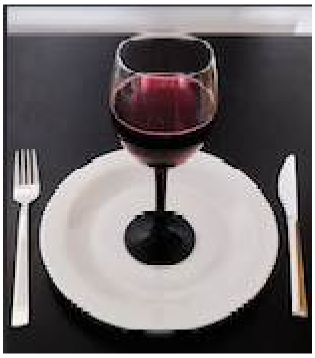

Zaznacz tylko jedną odpowiedź.

- Yes
- No

109. Do you think this meal is for you: *

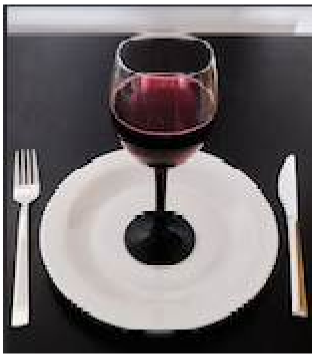

Zaznacz tylko jedną odpowiedź.

- too caloric
- has the right amount of kcal
- it has too few calories, I can or should eat a larger portion

110. How many calories do you think the meal in the photo has? *

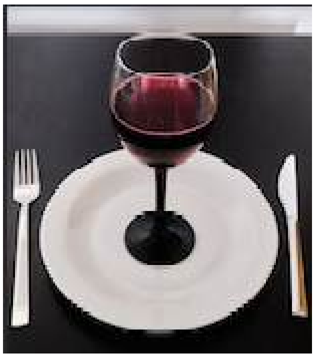

111. How long after eating the meal would you start feeling hungry again? *

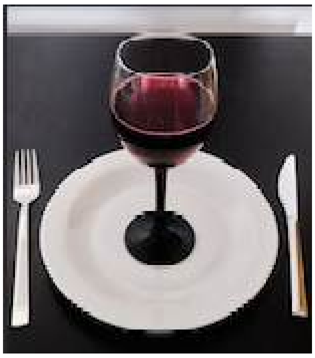

Zaznacz tylko jedną odpowiedź.

- Before the hour is up
- After 1 hour to 2 hours
- Over 2 hours, up to 3 hours
- Over 3 hours

Milk chocolate

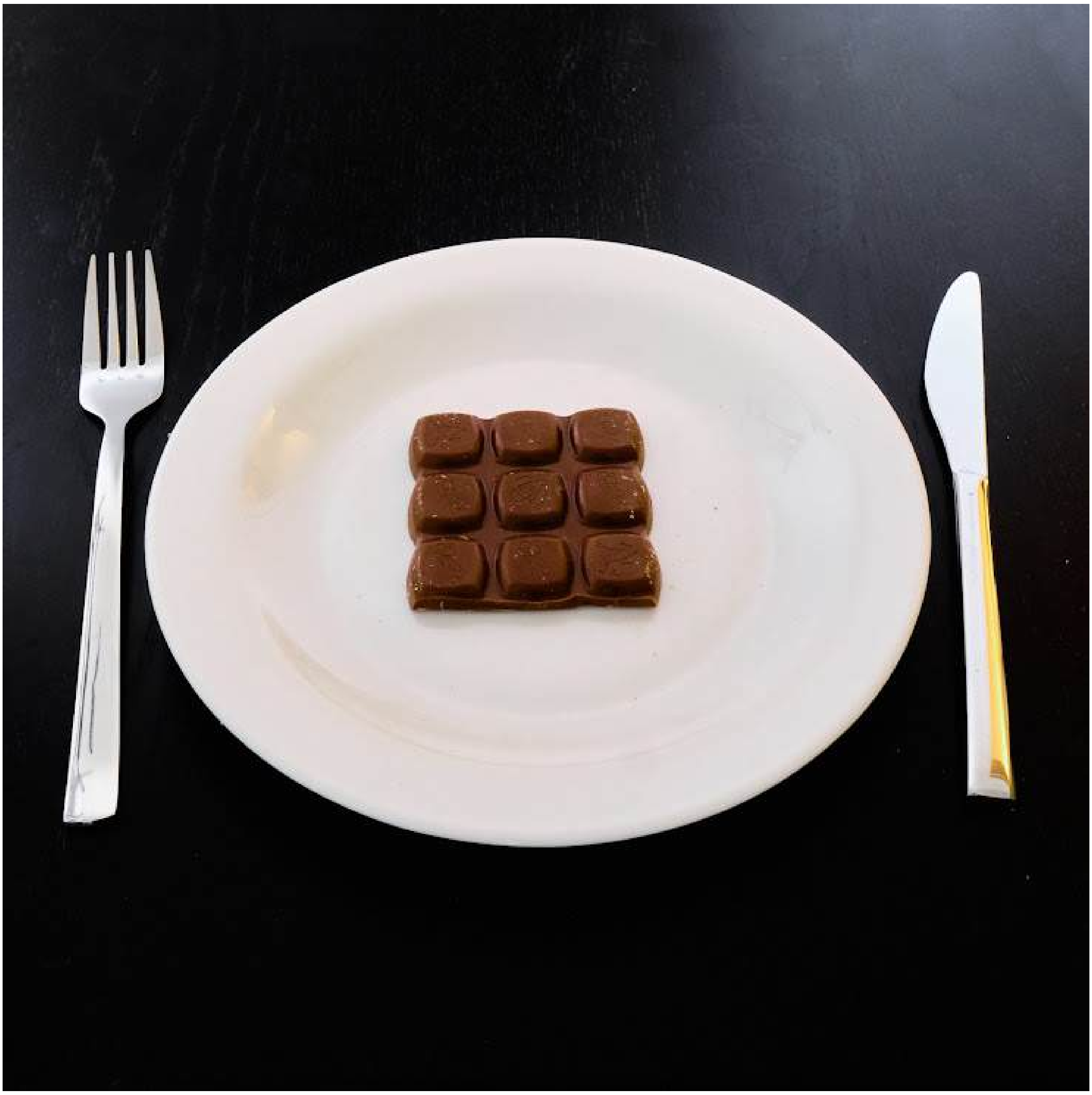

112. Determine the size of the meal shown in the photo *

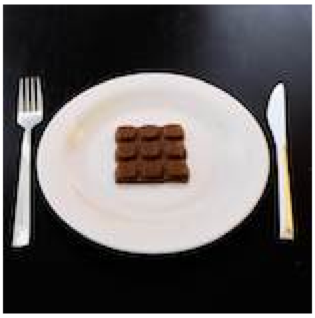

Zaznacz tylko jedną odpowiedź.

	Very small, almost nothing
1	◯
2	◯
3	◯
4	◯
5	◯
6	◯
7	◯
8	◯
9	◯
10	◯
	Very Large

113. How full would you be with this meal? *

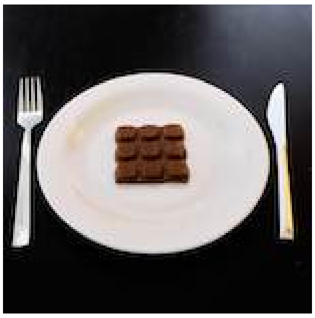

Zaznacz tylko jedną odpowiedź.

	Not full at all, still hungry
1	◯
2	◯
3	◯
4	◯
5	◯
6	◯
7	◯
8	◯
9	◯
10	◯
	Extremely full, on the verge of overeating

114. Do you think the meal in the photo is healthy? *

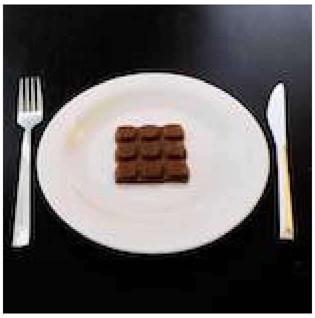

Zaznacz tylko jedną odpowiedź.

- Yes
- No

115. Would you still be hungry immediately after eating this meal? *

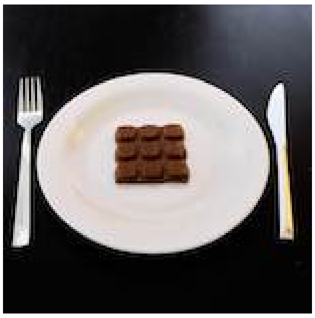

Zaznacz tylko jedną odpowiedź.

- Yes
- No

116. Do you think this meal is for you: *

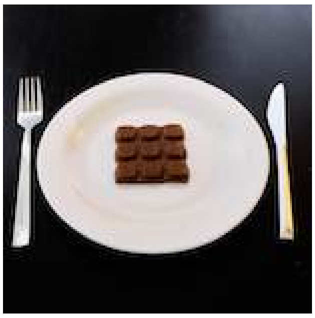

Zaznacz tylko jedną odpowiedź.

- too caloric
- has the right amount of kcal
- it has too few calories, I can or should eat a larger portion

117. How many calories do you think the meal in the photo has? *

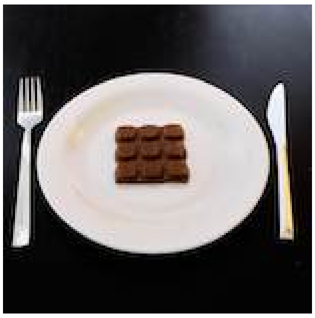

____

118. How long after eating the meal would you start feeling hungry again? *

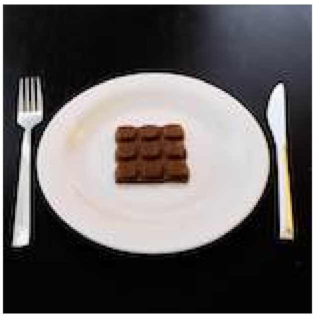

Zaznacz tylko jedną odpowiedź.

- Before the hour is up
- After 1 hour to 2 hours
- Over 2 hours, up to 3 hours
- Over 3 hours

A handful of walnuts

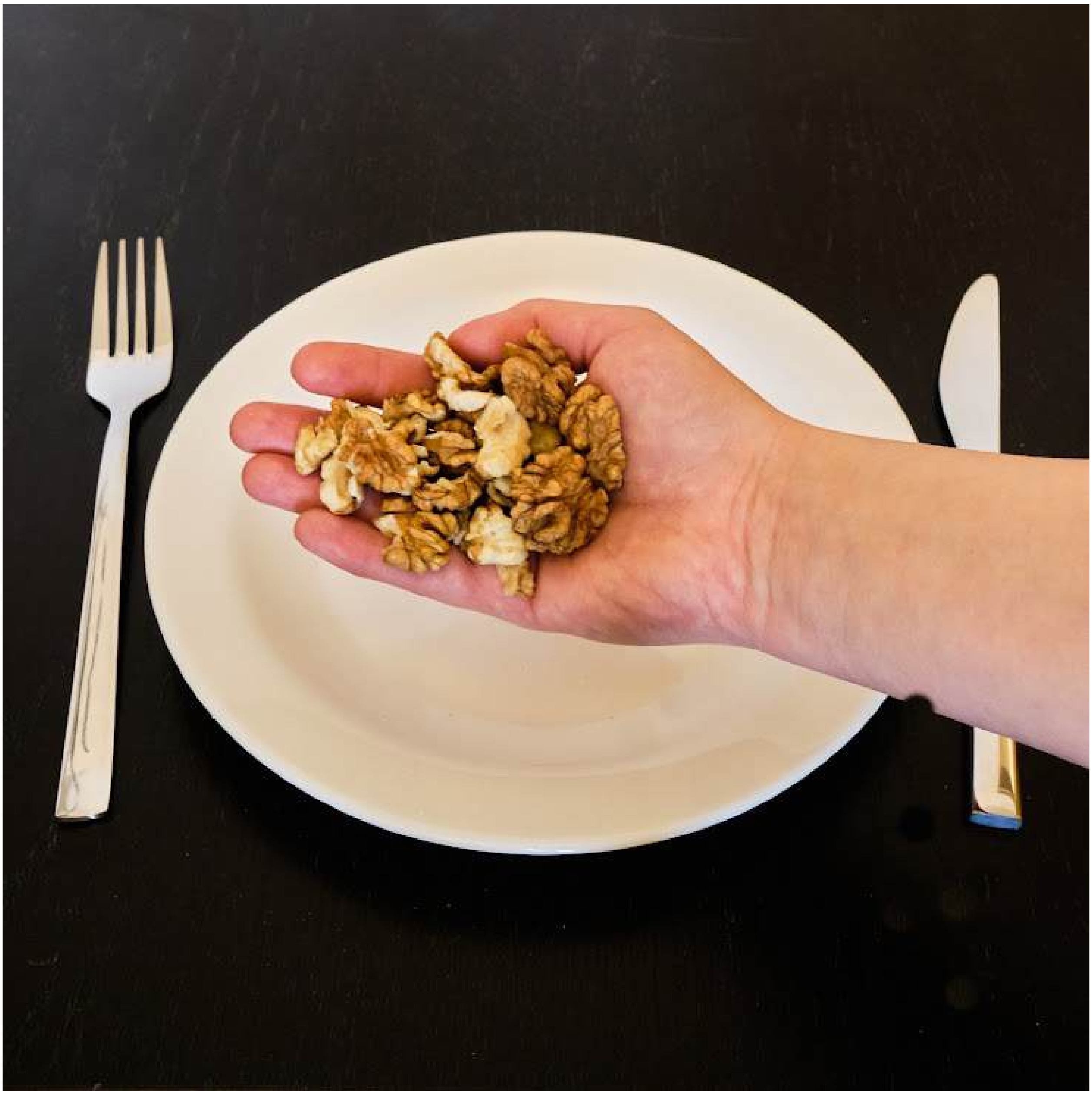

119. Determine the size of the meal shown in the photo *

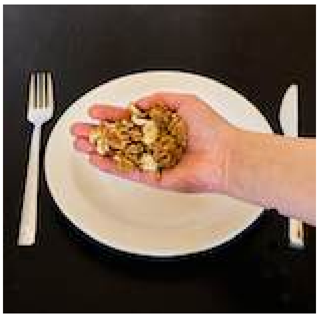

Zaznacz tylko jedną odpowiedź.

	Very small, almost nothing
1	◯
2	◯
3	◯
4	◯
5	◯
6	◯
7	◯
8	◯
9	◯
10	◯
	Very Large

120. How full would you be with this meal? *

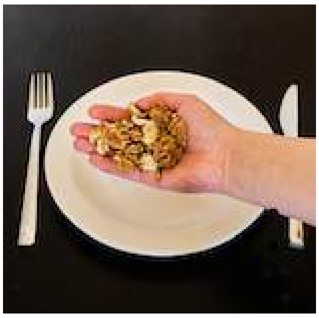

Zaznacz tylko jedną odpowiedź.

	Not full at all, still hungry
1	( )
2	( )
3	( )
4	( )
5	( )
6	( )
7	( )
8	( )
9	( )
10	( )
	Extremely full, on the verge of overeating

121. Do you think the meal in the photo is healthy? *

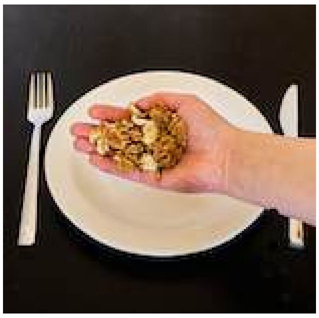

Zaznacz tylko jedną odpowiedź.

- Yes
- No

122. Would you still be hungry immediately after eating this meal? *

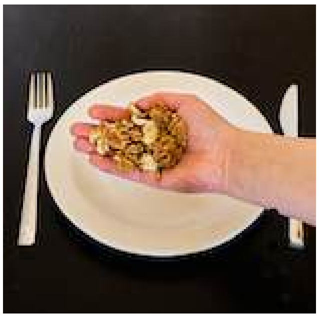

Zaznacz tylko jedną odpowiedź.

- Yes
- No

123. Do you think this meal is for you: *

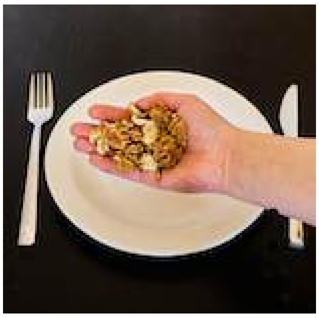

Zaznacz tylko jedną odpowiedź.

- too caloric
- has the right amount of kcal
- it has too few calories, I can or should eat a larger portion

124. How many calories do you think the meal in the photo has? *

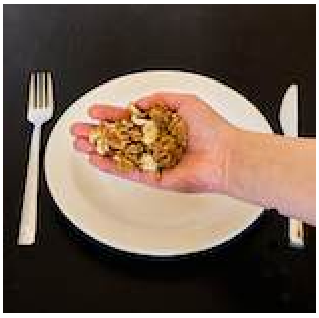

____

125. How long after eating the meal would you start feeling hungry again? *

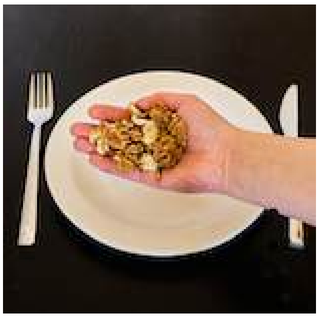

Zaznacz tylko jedną odpowiedź.

- Before the hour is up
- After 1 hour to 2 hours
- Over 2 hours, up to 3 hours
- Over 3 hours

Crispy cookies with cream filling

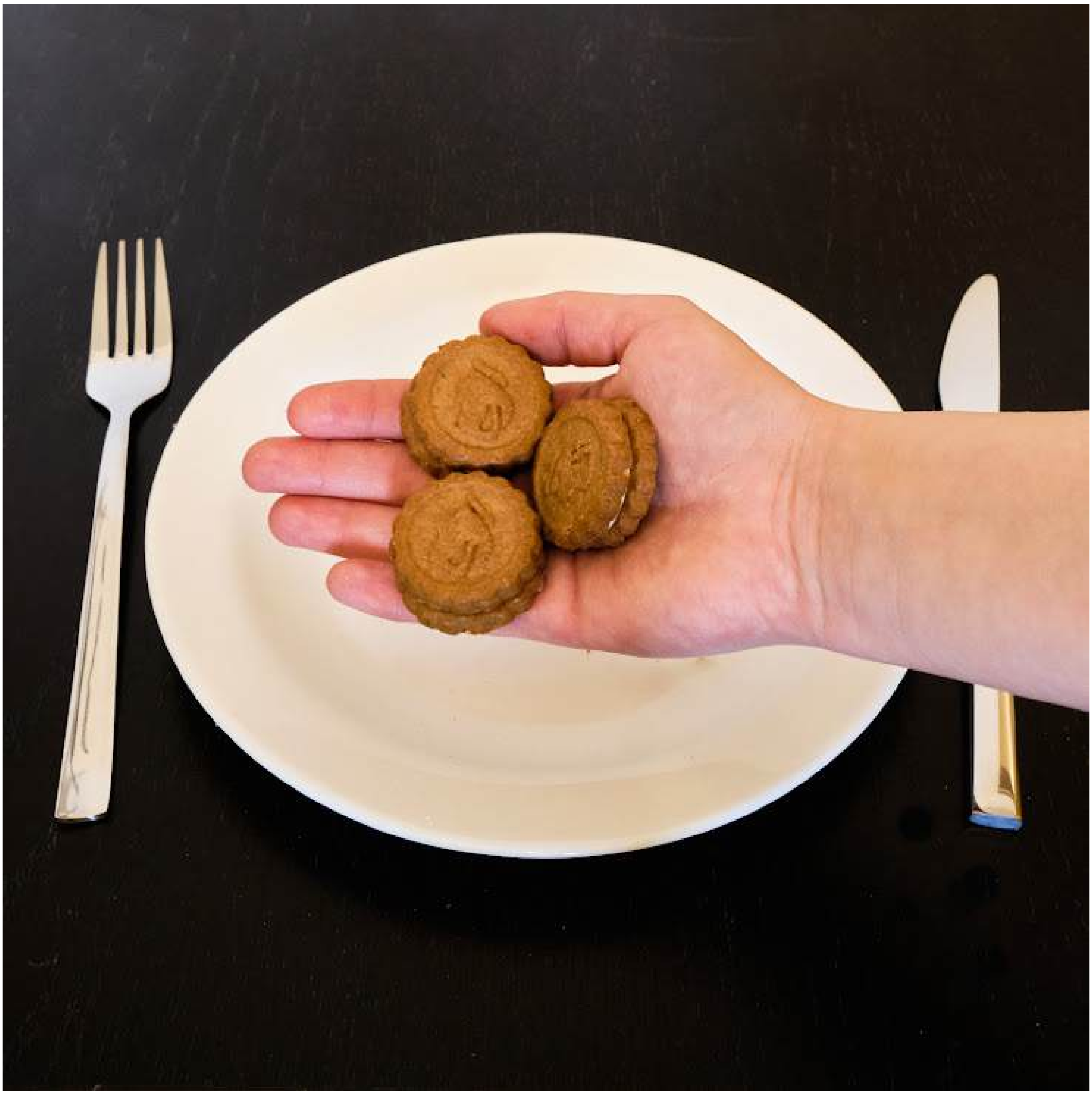

126. Determine the size of the meal shown in the photo *

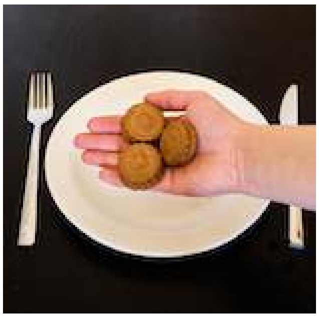

Zaznacz tylko jedną odpowiedź.

	Very small, almost nothing
1	◯
2	◯
3	◯
4	◯
5	◯
6	◯
7	◯
8	◯
9	◯
10	◯
	Very Large

127. How full would you be with this meal? *

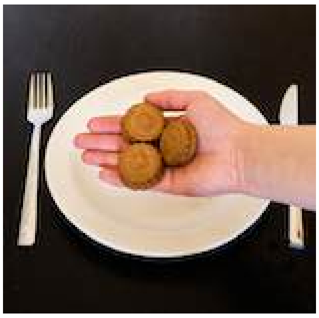

Zaznacz tylko jedną odpowiedź.

Not full at all, still hungry

1 ◯
2 ◯
3 ◯
4 ◯
5 ◯
6 ◯
7 ◯
8 ◯
9 ◯
10 ◯

Extremely full, on the verge of overeating

128. Do you think the meal in the photo is healthy? *

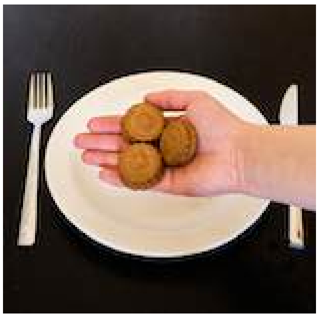

Zaznacz tylko jedną odpowiedź.

- Yes
- No

129. Would you still be hungry immediately after eating this meal? *

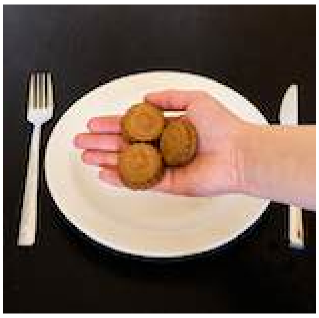

Zaznacz tylko jedną odpowiedź.

- Yes
- No

130. Do you think this meal is for you: *

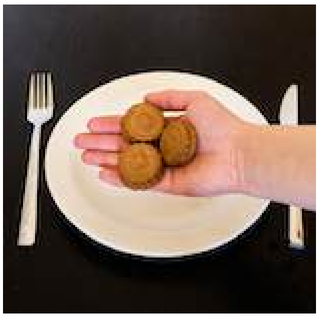

Zaznacz tylko jedną odpowiedź.

- too caloric
- has the right amount of kcal
- it has too few calories, I can or should eat a larger portion

131. How many calories do you think the meal in the photo has? *

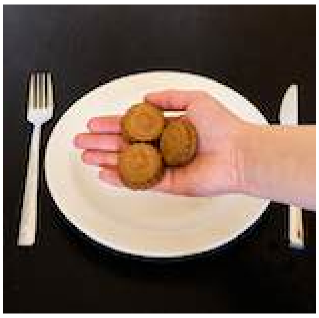

132. How long after eating the meal would you start feeling hungry again? *

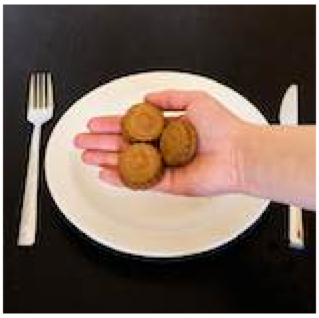

Zaznacz tylko jedną odpowiedź.

- Before the hour is up
- After 1 hour to 2 hours
- Over 2 hours, up to 3 hours
- Over 3 hours

Hot dog with mustard and pork sausage

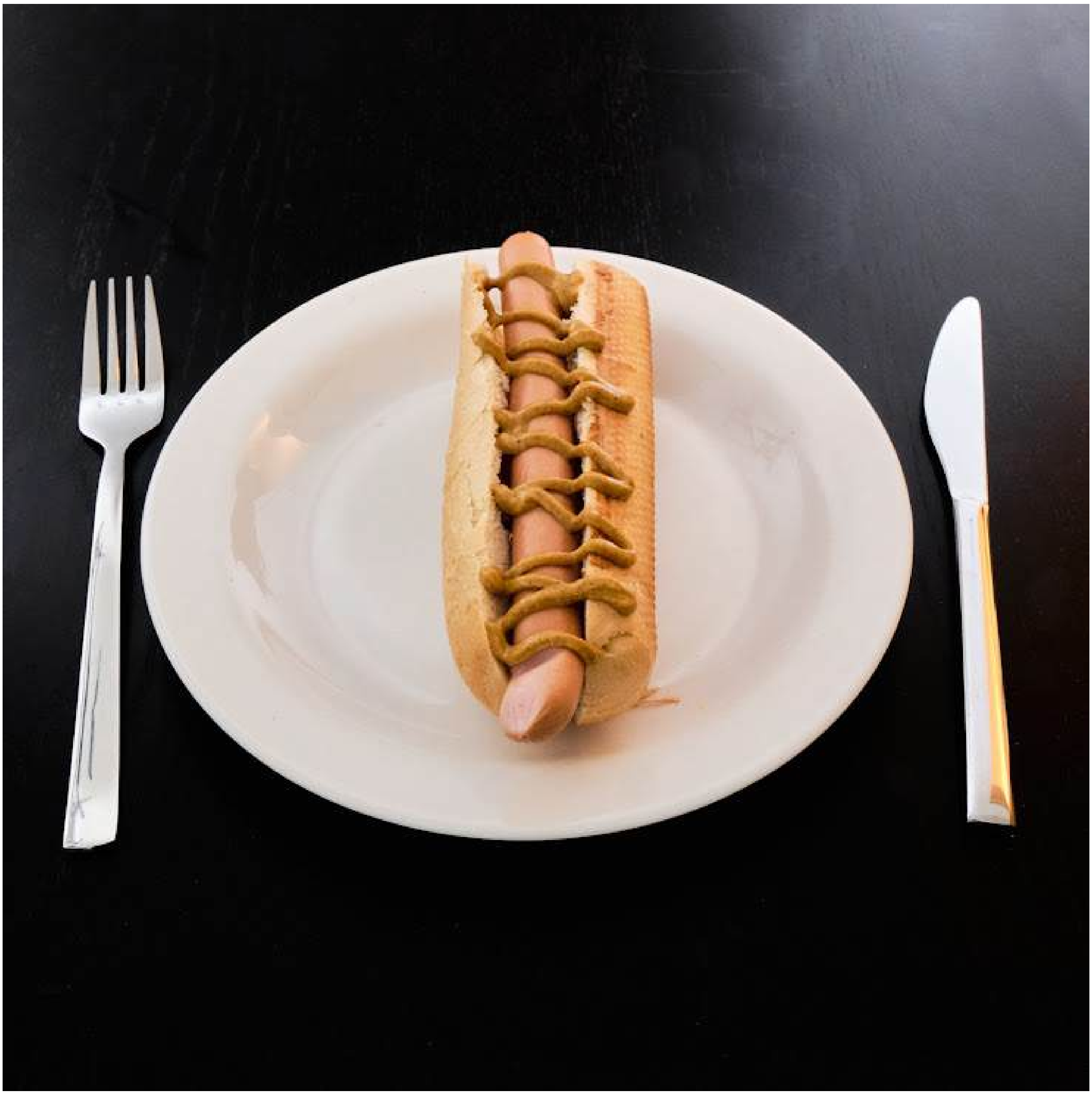

133. Determine the size of the meal shown in the photo *

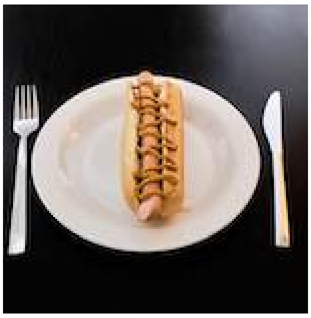

Zaznacz tylko jedną odpowiedź.

Very small, almost nothing
1
2
3
4
5
6
7
8
9
10
Very Large

134. How full would you be with this meal? *

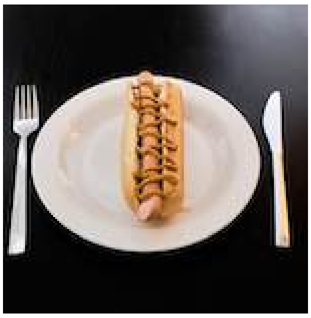

Zaznacz tylko jedną odpowiedź.

Not full at all, still hungry
1 ◯
2 ◯
3 ◯
4 ◯
5 ◯
6 ◯
7 ◯
8 ◯
9 ◯
10 ◯
Extremely full, on the verge of overeating

135. Do you think the meal in the photo is healthy? *

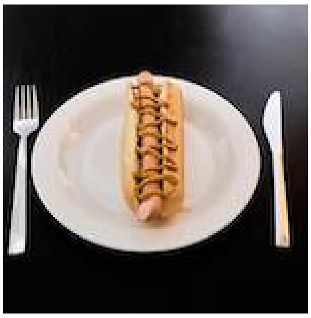

Zaznacz tylko jedną odpowiedź.

- Yes
- No

136. Would you still be hungry immediately after eating this meal? *

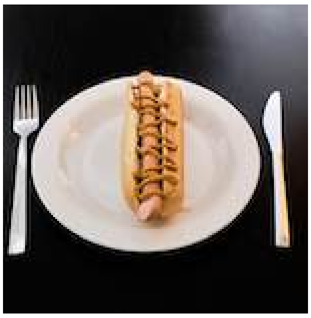

Zaznacz tylko jedną odpowiedź.

- Yes
- No

137. Do you think this meal is for you: *

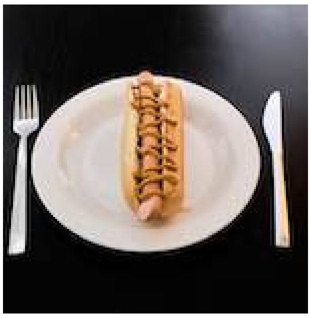

Zaznacz tylko jedną odpowiedź.

- too caloric
- has the right amount of kcal
- it has too few calories, I can or should eat a larger portion

138. How many calories do you think the meal in the photo has? *

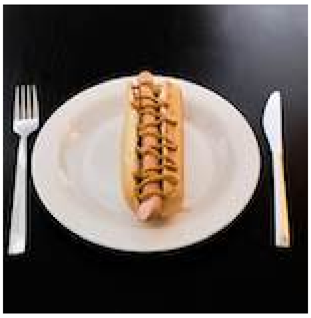

139. How long after eating the meal would you start feeling hungry again? *

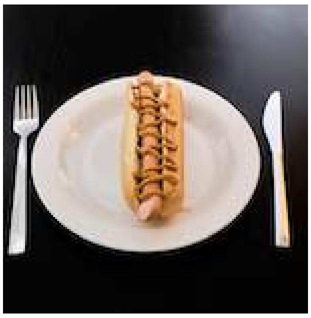

Zaznacz tylko jedną odpowiedź.

- Before the hour is up
- After 1 hour to 2 hours
- Over 2 hours, up to 3 hours
- Over 3 hours

Brown pasta with pesto, tomatoes and mozzarella

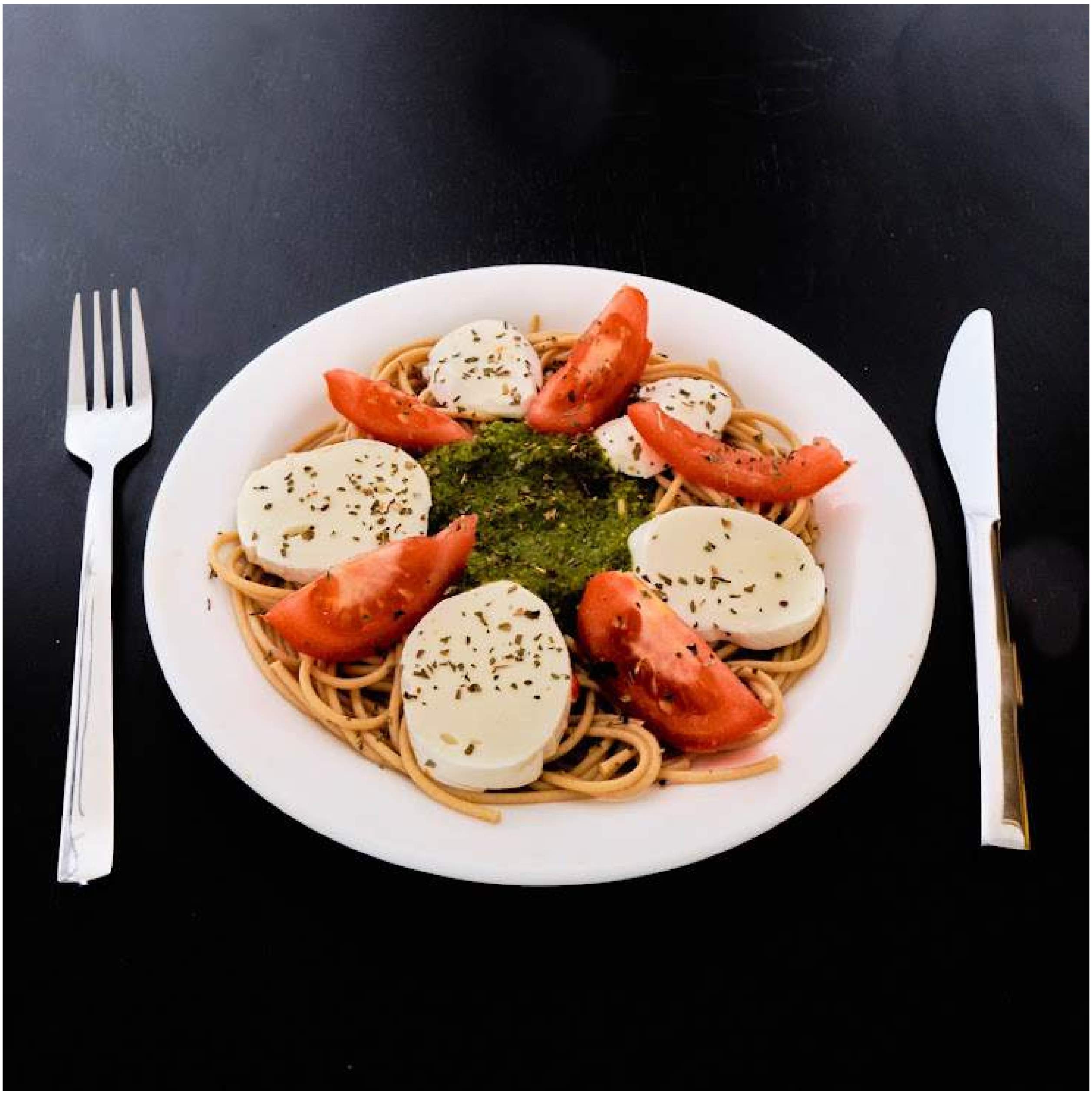

140. Determine the size of the meal shown in the photo *

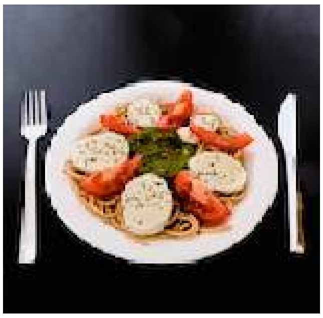

Zaznacz tylko jedną odpowiedź.

	Very small, almost nothing
1	⬭
2	⬭
3	⬭
4	⬭
5	⬭
6	⬭
7	⬭
8	⬭
9	⬭
10	⬭
	Very Large

141. How full would you be with this meal? *

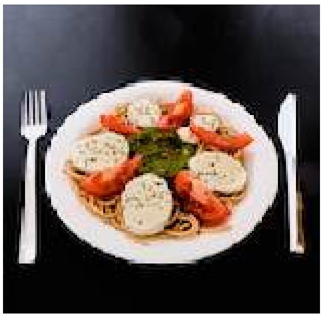

Zaznacz tylko jedną odpowiedź.

Not full at all, still hungry

1

2

3

4

5

6

7

8

9

10

Extremely full, on the verge of overeating

142. Do you think the meal in the photo is healthy? *

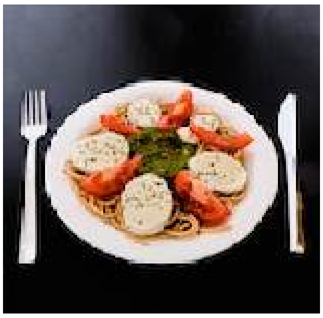

Zaznacz tylko jedną odpowiedź.

- Yes
- No

143. Would you still be hungry immediately after eating this meal? *

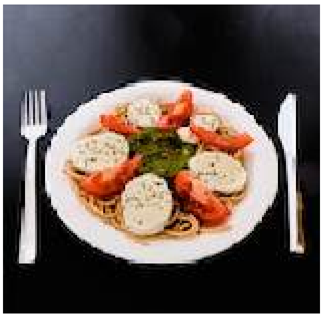

Zaznacz tylko jedną odpowiedź.

- Yes
- No

144. Do you think this meal is for you: *

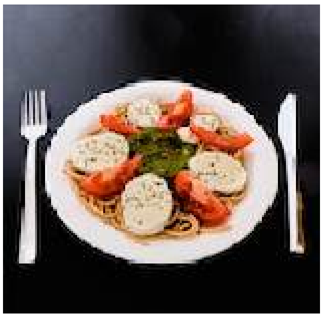

Zaznacz tylko jedną odpowiedź.

- too caloric
- has the right amount of kcal
- it has too few calories, I can or should eat a larger portion

145. How many calories do you think the meal in the photo has? *

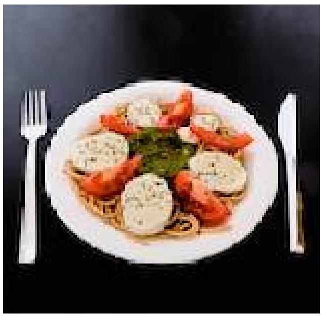

146. How long after eating the meal would you start feeling hungry again? *

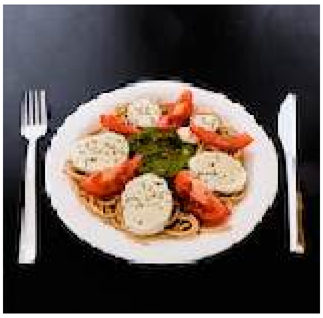

Zaznacz tylko jedną odpowiedź.

- Before the hour is up
- After 1 hour to 2 hours
- Over 2 hours, up to 3 hours
- Over 3 hours

Mini carrots

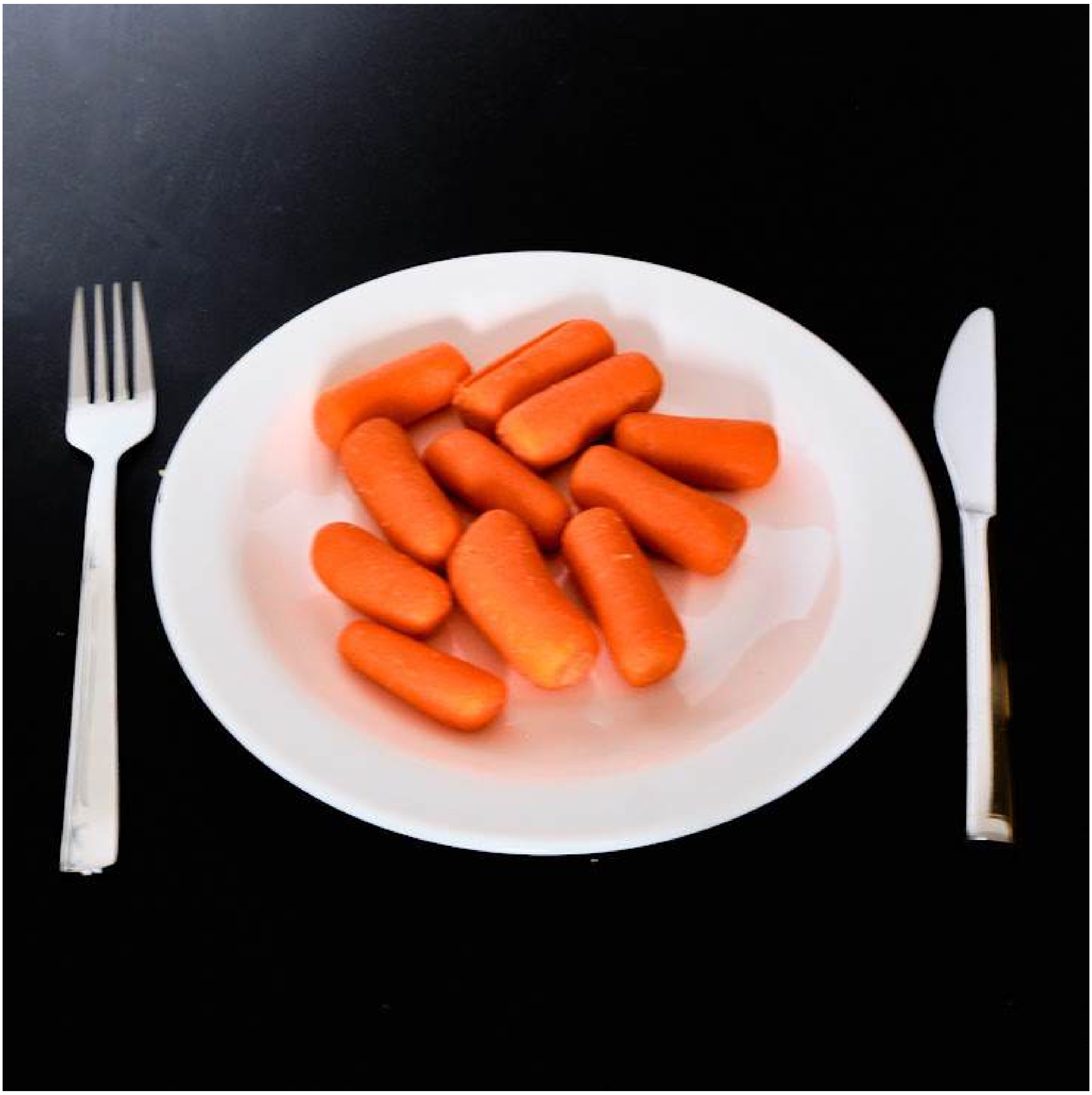

147. Determine the size of the meal shown in the photo *

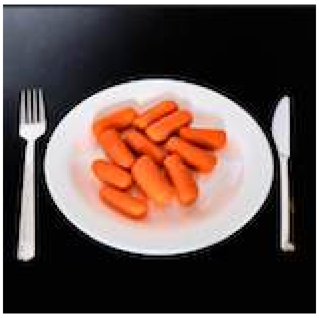

Zaznacz tylko jedną odpowiedź.

	Very small, almost nothing
1	◯
2	◯
3	◯
4	◯
5	◯
6	◯
7	◯
8	◯
9	◯
10	◯
	Very Large

148. How full would you be with this meal? *

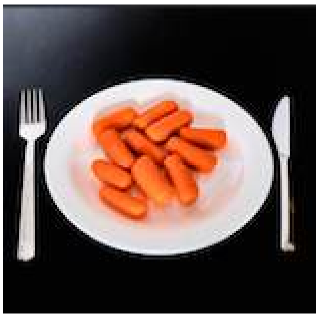

Zaznacz tylko jedną odpowiedź.

	Not full at all, still hungry
1	◯
2	◯
3	◯
4	◯
5	◯
6	◯
7	◯
8	◯
9	◯
10	◯
	Extremely full, on the verge of overeating

149. Do you think the meal in the photo is healthy? *

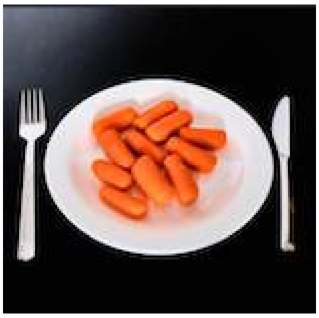

Zaznacz tylko jedną odpowiedź.

- Yes
- No

150. Would you still be hungry immediately after eating this meal? *

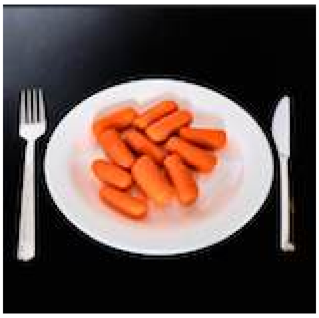

Zaznacz tylko jedną odpowiedź.

- Yes
- No

151. Do you think this meal is for you: *

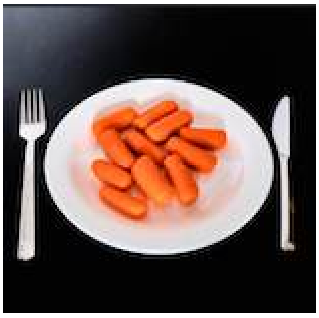

Zaznacz tylko jedną odpowiedź.

- too caloric
- has the right amount of kcal
- it has too few calories, I can or should eat a larger portion

152. How many calories do you think the meal in the photo has? *

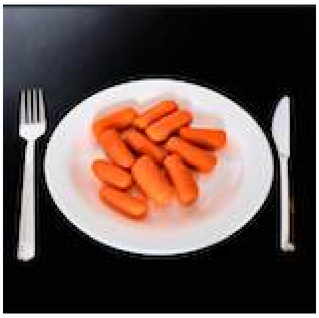

____

153. long *

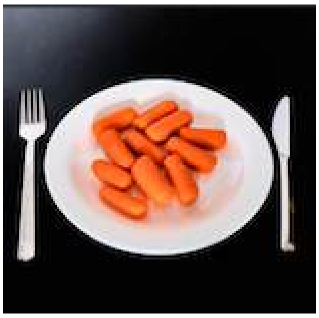

Zaznacz tylko jedną odpowiedź.

- Before the hour is up
- After 1 hour to 2 hours
- Over 2 hours, up to 3 hours
- Over 3 hours

Creamy tomato soup (with natural yogurt, a spoon of olive oil)

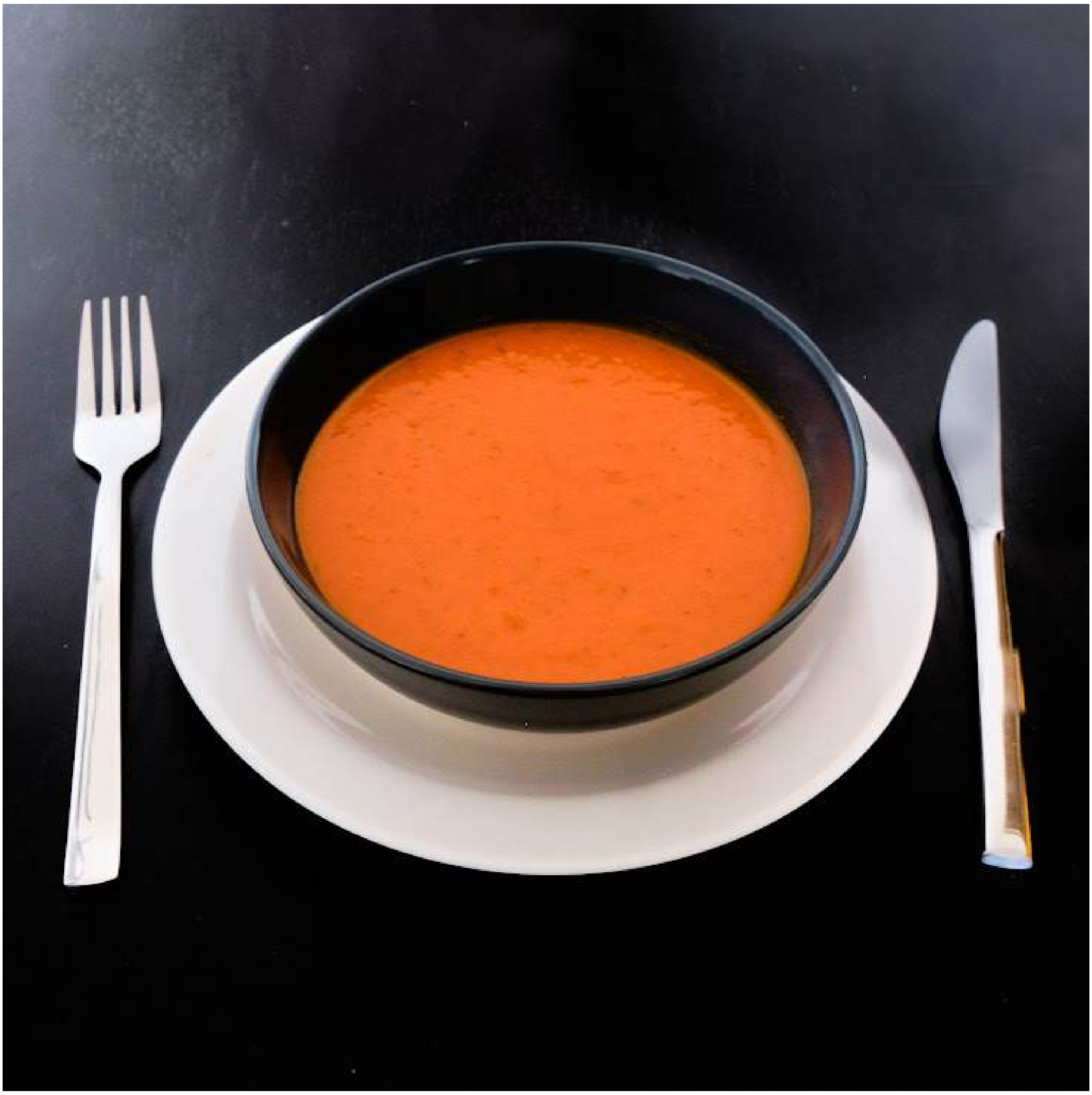

154. Determine the size of the meal shown in the photo *

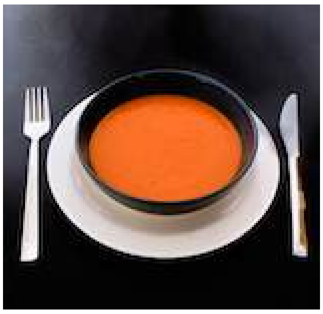

Zaznacz tylko jedną odpowiedź.

Very small, almost nothing

1

2

3

4

5

6

7

8

9

10

Very Large

155. How full would you be with this meal? *

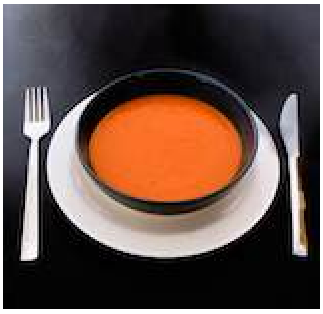

Zaznacz tylko jedną odpowiedź.

Not full at all, still hungry
1 ◯
2 ◯
3 ◯
4 ◯
5 ◯
6 ◯
7 ◯
8 ◯
9 ◯
10 ◯
Extremely full, on the verge of overeating

156. Do you think the meal in the photo is healthy? *

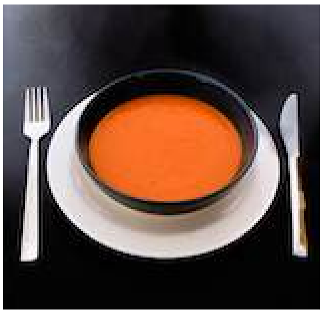

Zaznacz tylko jedną odpowiedź.

- Yes
- No

157. Would you still be hungry immediately after eating this meal? *

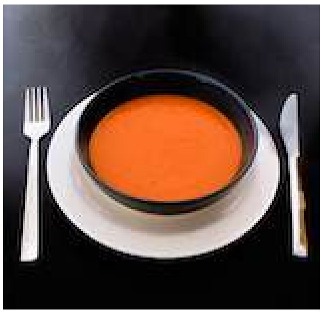

Zaznacz tylko jedną odpowiedź.

- Yes
- No

158. Do you think this meal is for you: *

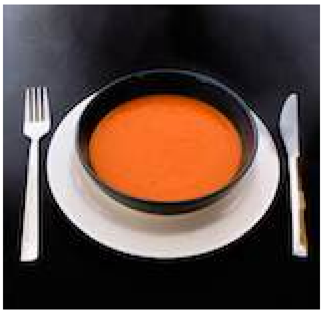

Zaznacz tylko jedną odpowiedź.

- too caloric
- has the right amount of kcal
- it has too few calories, I can or should eat a larger portion

159. How many calories do you think the meal in the photo has? *

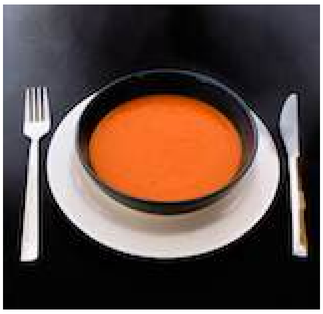

160. How long after eating the meal would you start feeling hungry again? *

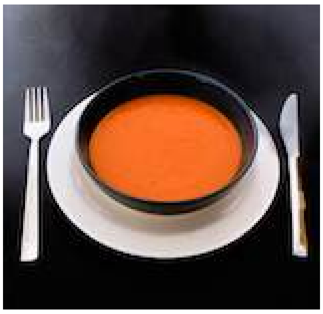

Zaznacz tylko jedną odpowiedź.

- Before the hour is up
- After 1 hour to 2 hours
- Over 2 hours, up to 3 hours
- Over 3 hours

OCENA WYBRANYCH POSIŁKÓW 3/3

The products are always on the same plate and the photos are taken from the same perspective. There is cutlery nearby to help you estimate portions.

Beer

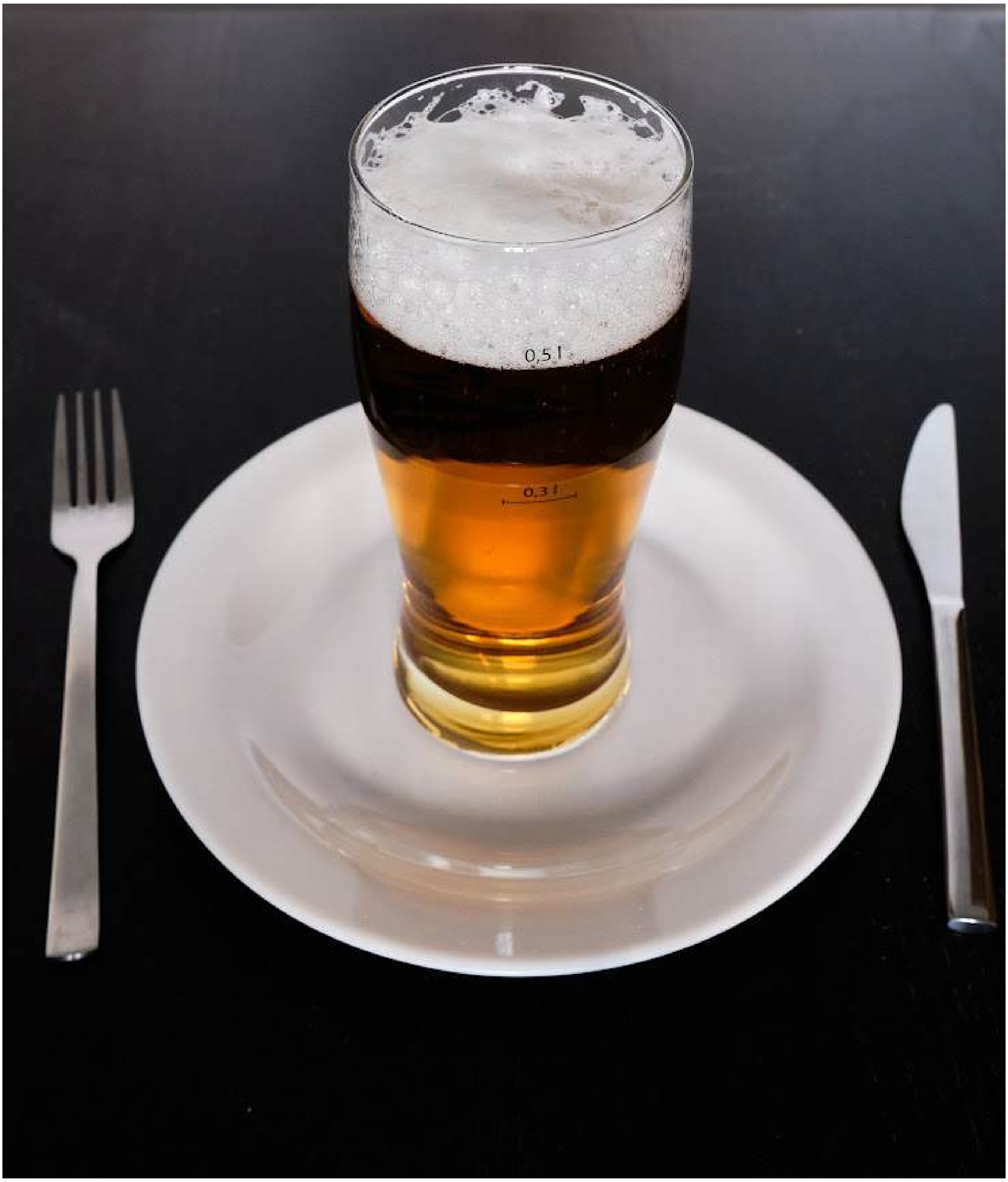

161. Determine the size of the meal shown in the photo *

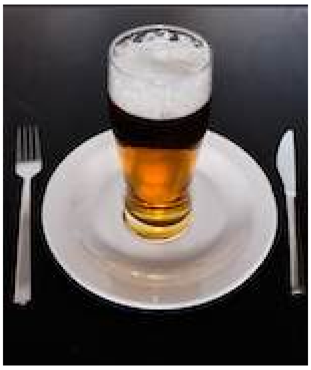

Zaznacz tylko jedną odpowiedź.

Very small, almost nothing

1 ◯

2 ◯

3 ◯

4 ◯

5 ◯

6 ◯

7 ◯

8 ◯

9 ◯

10 ◯

Very Large

162. How full would you be with this meal? *

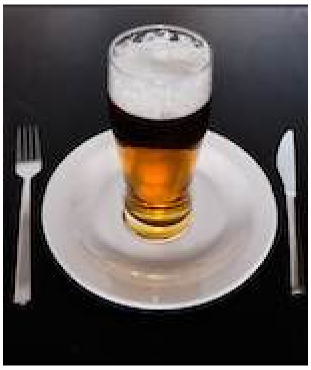

Zaznacz tylko jedną odpowiedź.

Not full at all, still hungry

1

2

3

4

5

6

7

8

9

10

Extremely full, on the verge of overeating

163. Do you think the meal in the photo is healthy? *

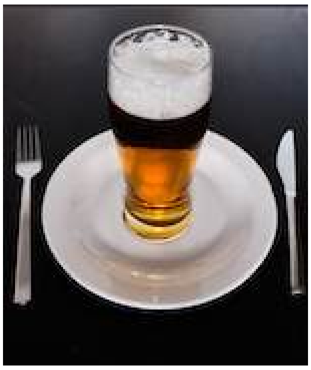

Zaznacz tylko jedną odpowiedź.

- Yes
- No

164. Would you still be hungry immediately after eating this meal? *

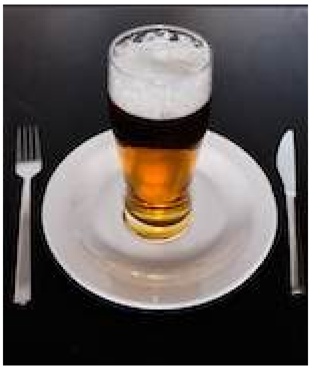

Zaznacz tylko jedną odpowiedź.

- Yes
- No

165. Do you think this meal is for you: *

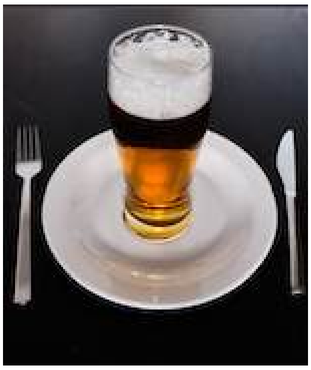

Zaznacz tylko jedną odpowiedź.

- too caloric
- has the right amount of kcal
- it has too few calories, I can or should eat a larger portion

166. How many calories do you think the meal in the photo has? *

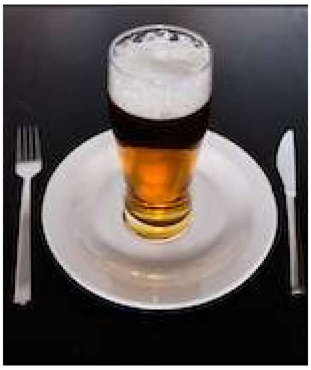

167. How long after eating the meal would you start feeling hungry again? *

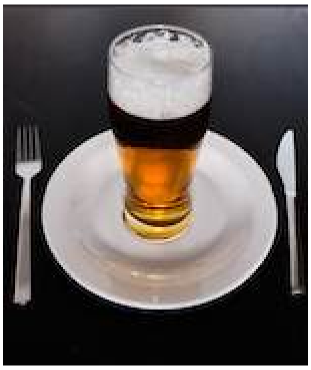

Zaznacz tylko jedną odpowiedź.

- Before the hour is up
- After 1 hour to 2 hours
- Over 2 hours, up to 3 hours
- Over 3 hours

Donut with rose filling

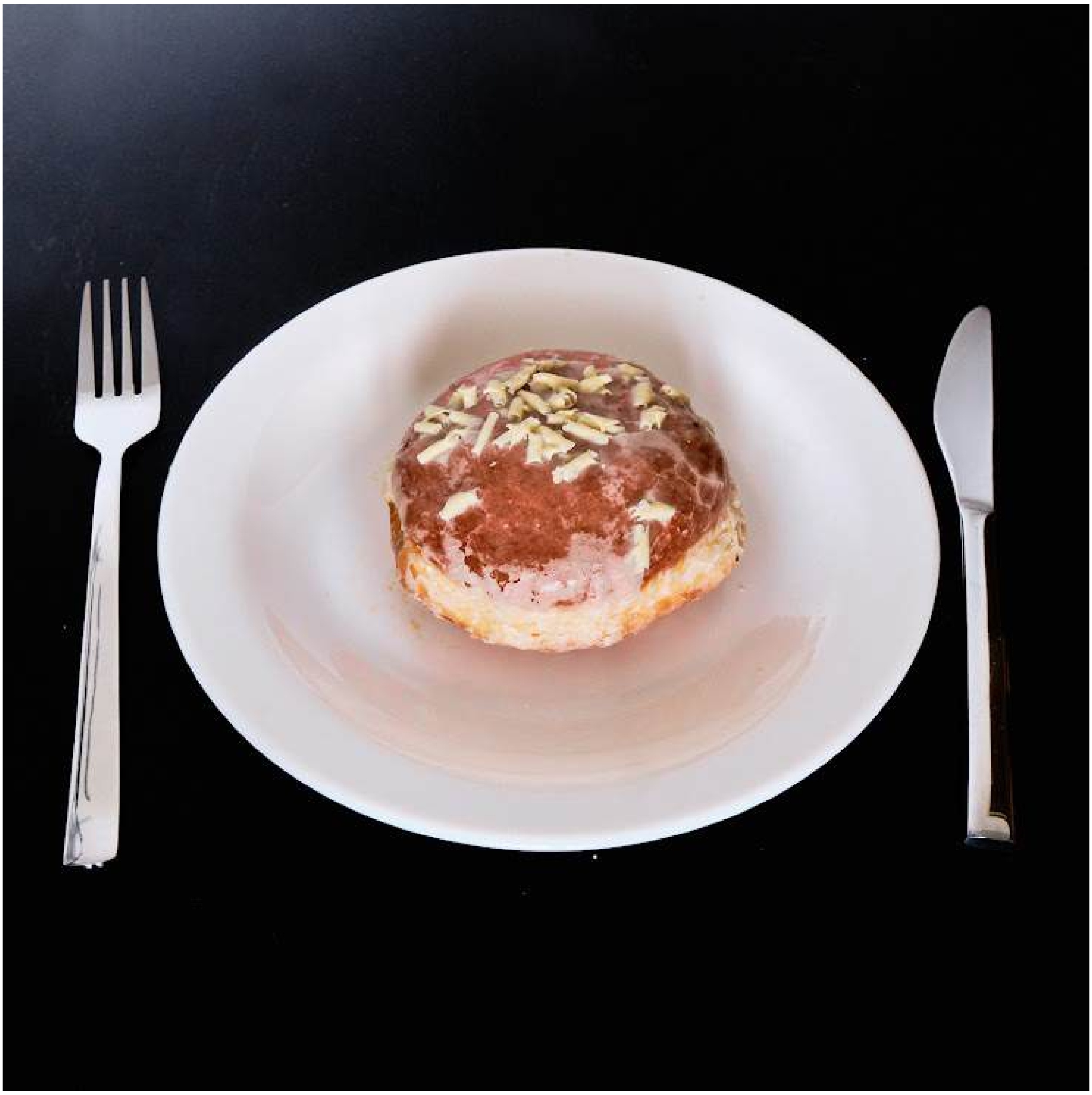

168. Determine the size of the meal shown in the photo *

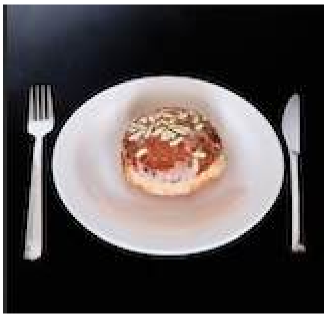

Zaznacz tylko jedną odpowiedź.

	Very small, almost nothing
1	⬭
2	⬭
3	⬭
4	⬭
5	⬭
6	⬭
7	⬭
8	⬭
9	⬭
10	⬭
	Very Large

169. How full would you be with this meal? *

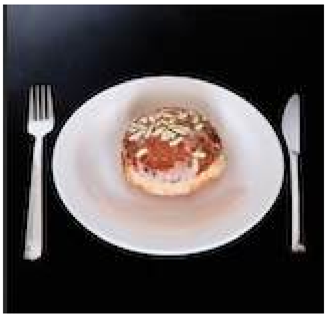

Zaznacz tylko jedną odpowiedź.

Not full at all, still hungry

1 ⬭

2 ⬭

3 ⬭

4 ⬭

5 ⬭

6 ⬭

7 ⬭

8 ⬭

9 ⬭

10 ⬭

Extremely full, on the verge of overeating

170. Do you think the meal in the photo is healthy? *

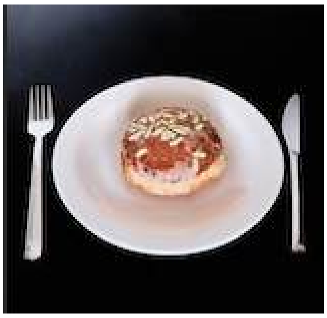

Zaznacz tylko jedną odpowiedź.

- Yes
- No

171. Would you still be hungry immediately after eating this meal? *

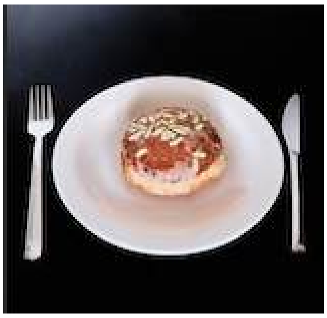

Zaznacz tylko jedną odpowiedź.

- Yes
- No

172. Do you think this meal is for you: *

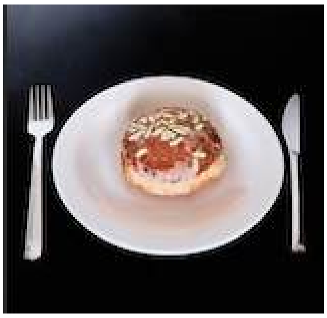

Zaznacz tylko jedną odpowiedź.

- too caloric
- has the right amount of kcal
- it has too few calories, I can or should eat a larger portion

173. How many calories do you think the meal in the photo has? *

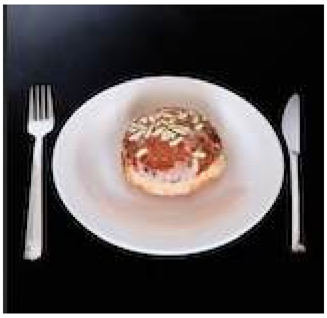

174. How long after eating the meal would you start feeling hungry again? *

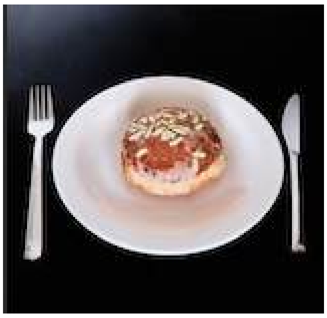

Zaznacz tylko jedną odpowiedź.

- Before the hour is up
- After 1 hour to 2 hours
- Over 2 hours, up to 3 hours
- Over 3 hours

Eggs fried in olive oil (1 tablespoon), Parma ham and avocado

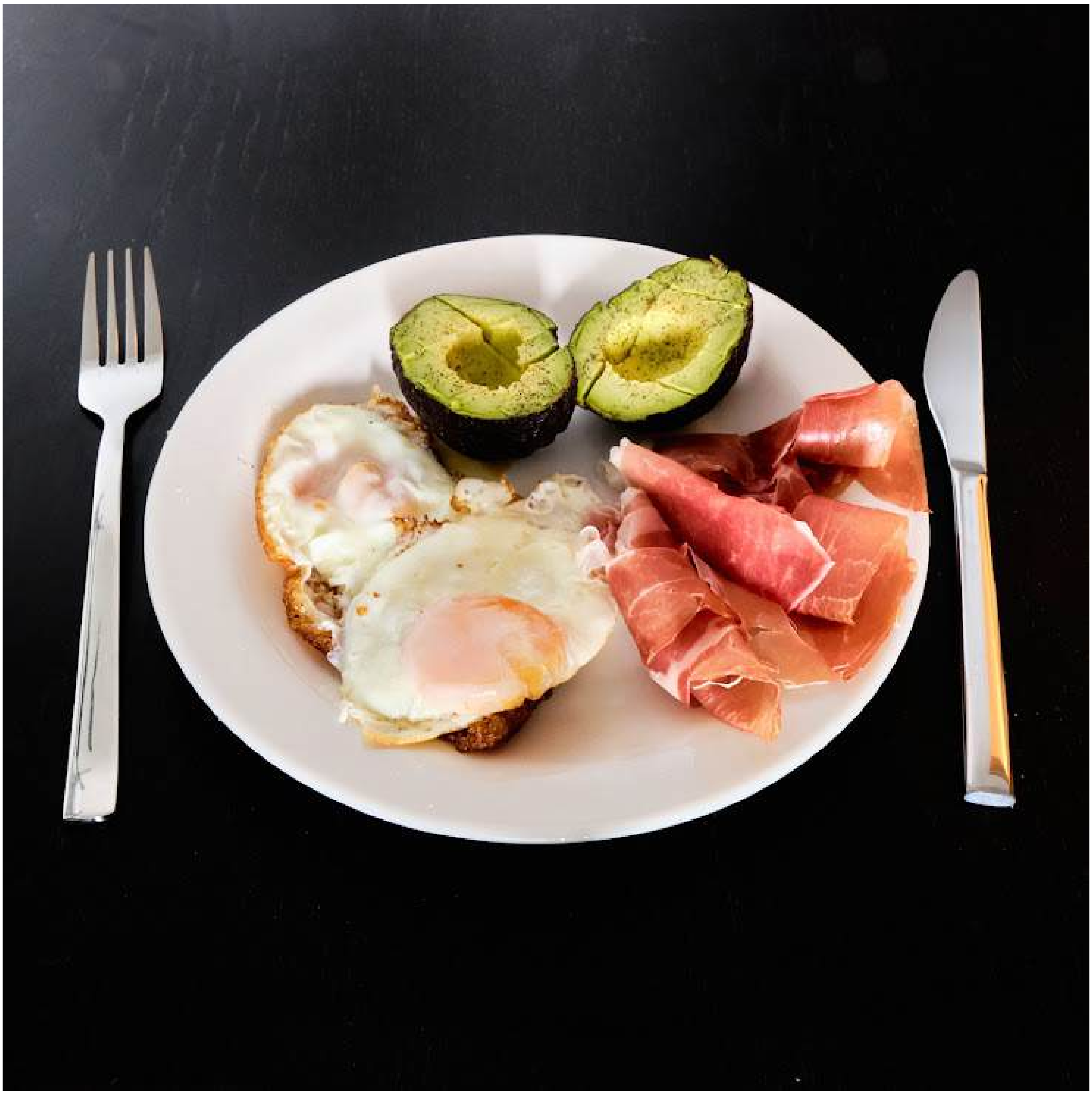

175. Determine the size of the meal shown in the photo *

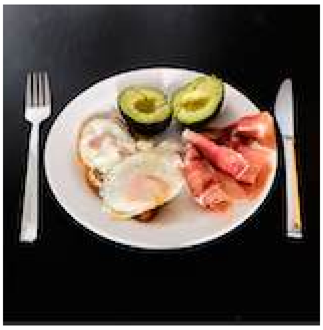

Zaznacz tylko jedną odpowiedź.

	Very small, almost nothing
1	◯
2	◯
3	◯
4	◯
5	◯
6	◯
7	◯
8	◯
9	◯
10	◯
	Very Large

176. How full would you be with this meal? *

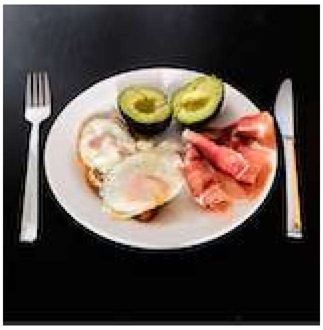

Zaznacz tylko jedną odpowiedź.

	Not full at all, still hungry
1	◯
2	◯
3	◯
4	◯
5	◯
6	◯
7	◯
8	◯
9	◯
10	◯
	Extremely full, on the verge of overeating

177. Do you think the meal in the photo is healthy? *

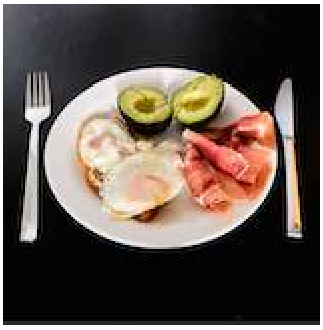

Zaznacz tylko jedną odpowiedź.

- Yes
- No

178. Would you still be hungry immediately after eating this meal? *

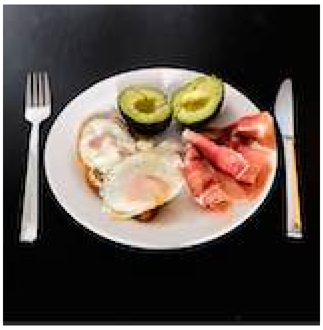

Zaznacz tylko jedną odpowiedź.

- Yes
- No

179. Do you think this meal is for you: *

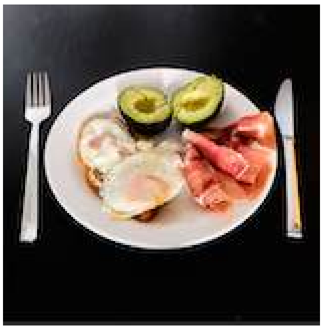

Zaznacz tylko jedną odpowiedź.

- too caloric
- has the right amount of kcal
- it has too few calories, I can or should eat a larger portion

180. How many calories do you think the meal in the photo has? *

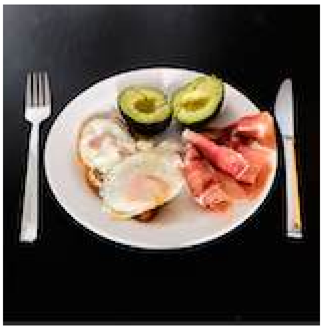

181. How long after eating the meal would you start feeling hungry again? *

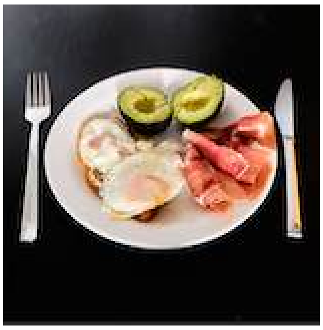

Zaznacz tylko jedną odpowiedź.

- Before the hour is up
- After 1 hour to 2 hours
- Over 2 hours, up to 3 hours
- Over 3 hours

Porridge (mountain oat flakes, raisins, nuts, natural yogurt)

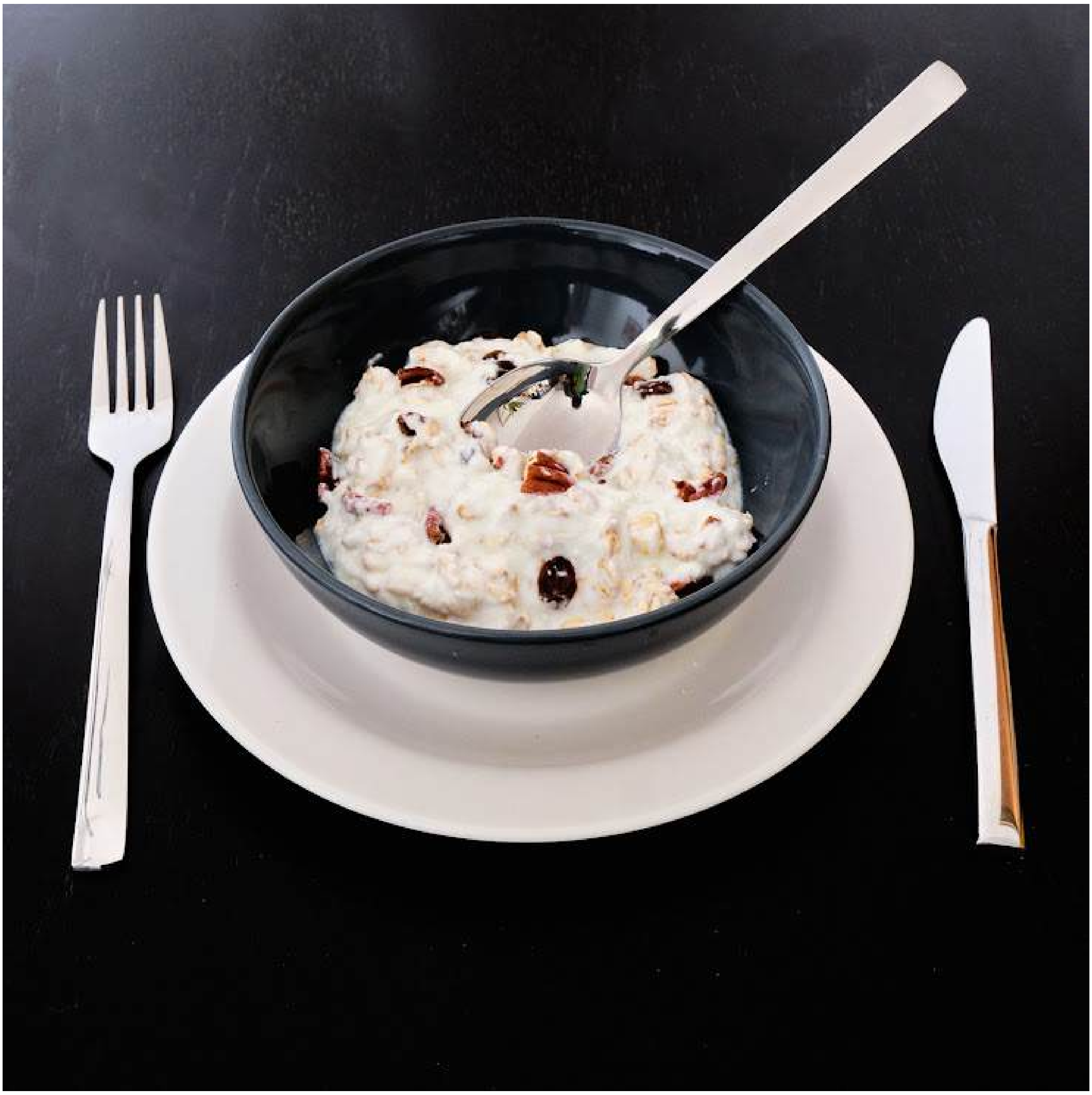

182. Determine the size of the meal shown in the photo *

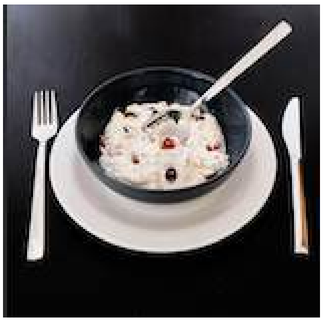

Zaznacz tylko jedną odpowiedź.

Very small, almost nothing

1 ◯

2 ◯

3 ◯

4 ◯

5 ◯

6 ◯

7 ◯

8 ◯

9 ◯

10 ◯

Very Large

183. How full would you be with this meal? *

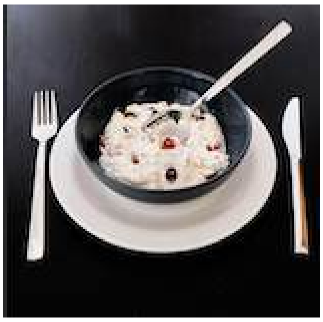

Zaznacz tylko jedną odpowiedź.

Not full at all, still hungry	
1	◯
2	◯
3	◯
4	◯
5	◯
6	◯
7	◯
8	◯
9	◯
10	◯
Extremely full, on the verge of overeating	

184. Do you think the meal in the photo is healthy? *

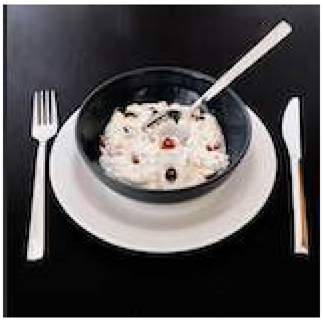

Zaznacz tylko jedną odpowiedź.

- Yes
- No

185. Would you still be hungry immediately after eating this meal? *

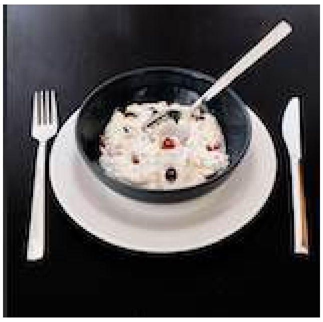

Zaznacz tylko jedną odpowiedź.

- Yes
- No

186. Do you think this meal is for you: *

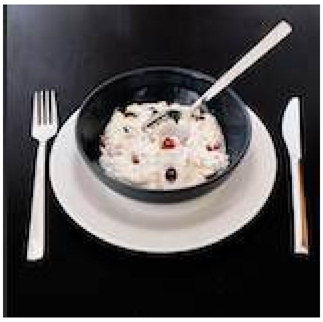

Zaznacz tylko jedną odpowiedź.

- too caloric
- has the right amount of kcal
- it has too few calories, I can or should eat a larger portion

187. How many calories do you think the meal in the photo has? *

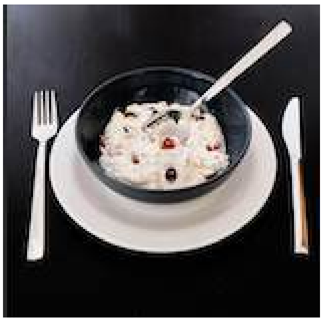

188. How long after eating the meal would you start feeling hungry again? *

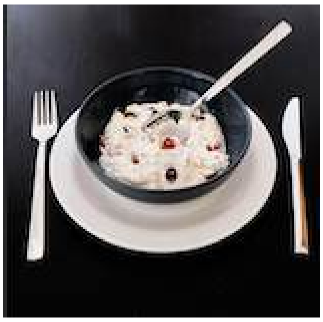

Zaznacz tylko jedną odpowiedź.

- Before the hour is up
- After 1 hour to 2 hours
- Over 2 hours, up to 3 hours
- Over 3 hours

Coca-cola

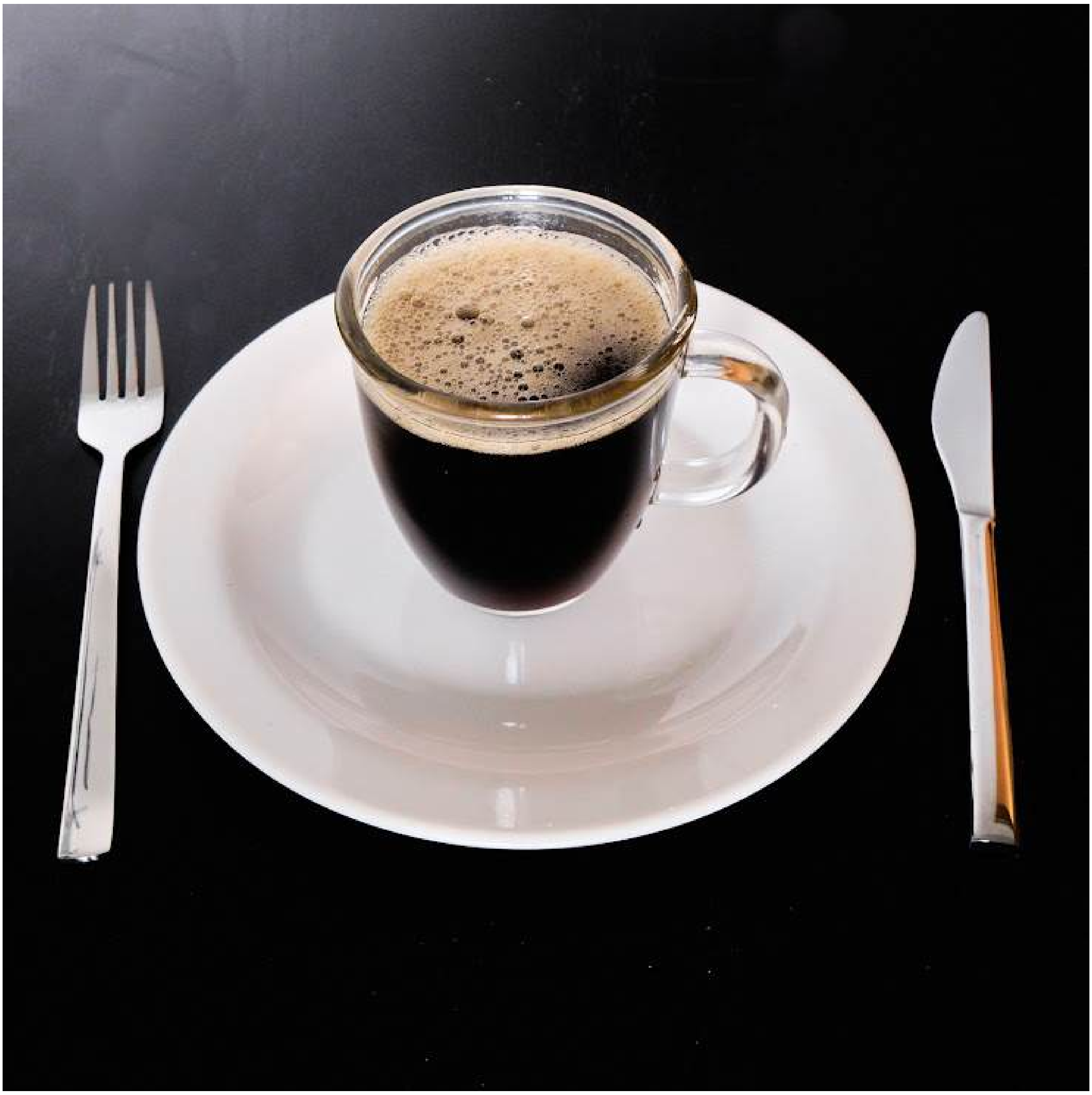

189. Determine the size of the meal shown in the photo *

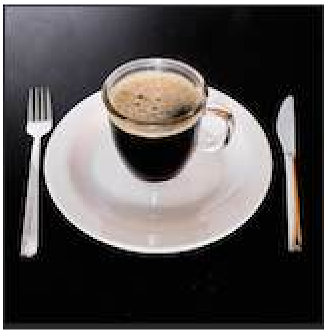

Zaznacz tylko jedną odpowiedź.

Very small, almost nothing

1

2

3

4

5

6

7

8

9

10

Very Large

190. How full would you be with this meal? *

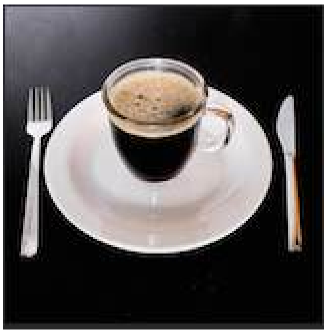

Zaznacz tylko jedną odpowiedź.

	Not full at all, still hungry
1	☐
2	☐
3	☐
4	☐
5	☐
6	☐
7	☐
8	☐
9	☐
10	☐
	Extremely full, on the verge of overeating

191. Do you think the meal in the photo is healthy? *

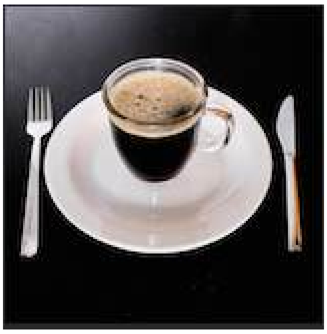

Zaznacz tylko jedną odpowiedź.

- Yes
- No

192. Would you still be hungry immediately after eating this meal? *

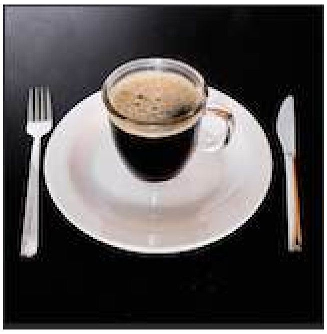

Zaznacz tylko jedną odpowiedź.

- Yes
- No

193. Do you think this meal is for you: *

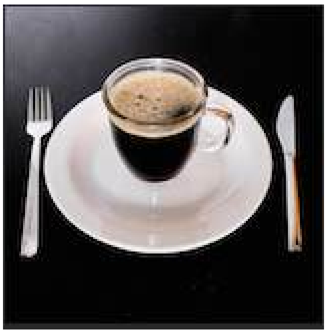

Zaznacz tylko jedną odpowiedź.

- too caloric
- has the right amount of kcal
- it has too few calories, I can or should eat a larger portion

194. How many calories do you think the meal in the photo has? *

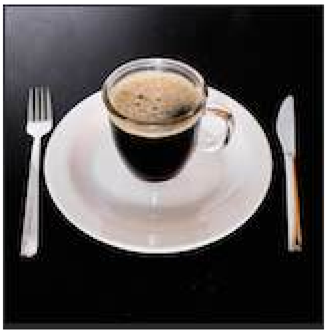

195. How long after eating the meal would you start feeling hungry again? *

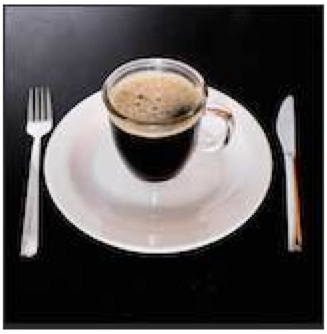

Zaznacz tylko jedną odpowiedź.

- Before the hour is up
- After 1 hour to 2 hours
- Over 2 hours, up to 3 hours
- Over 3 hours

Apple

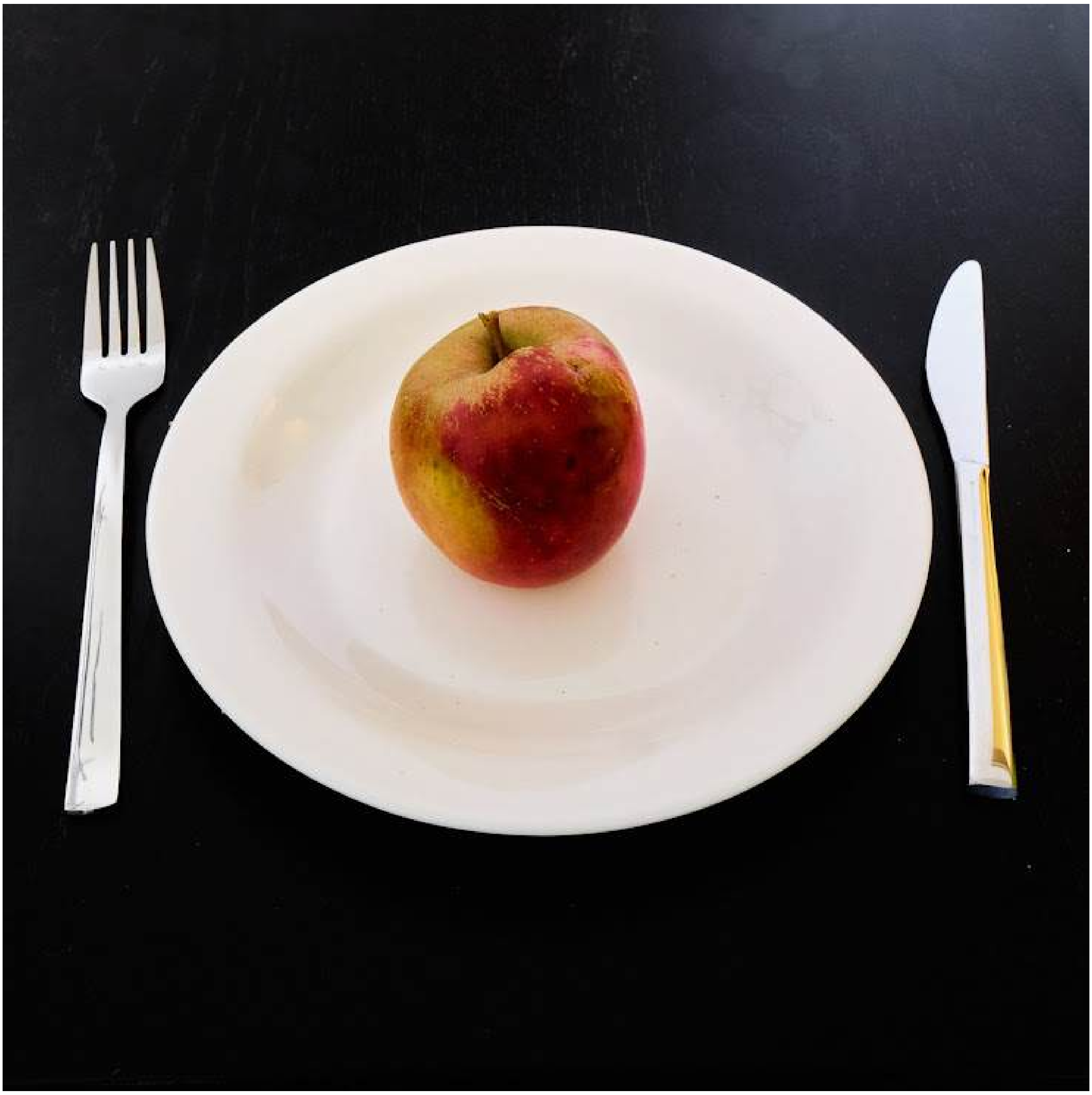

196. Determine the size of the meal shown in the photo *

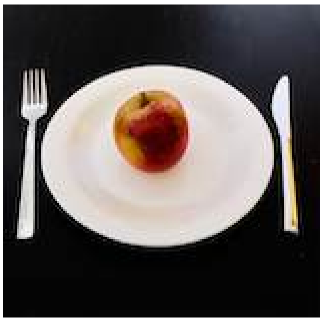

Zaznacz tylko jedną odpowiedź.

Very small, almost nothing

1 ☐

2 ☐

3 ☐

4 ☐

5 ☐

6 ☐

7 ☐

8 ☐

9 ☐

10 ☐

Very Large

197. How full would you be with this meal? *

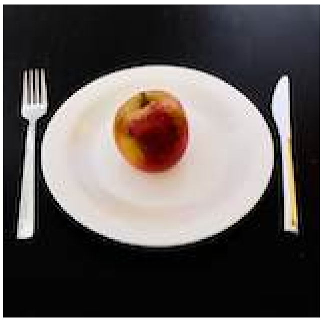

Zaznacz tylko jedną odpowiedź.

	Not full at all, still hungry
1	⬭
2	⬭
3	⬭
4	⬭
5	⬭
6	⬭
7	⬭
8	⬭
9	⬭
10	⬭
	Extremely full, on the verge of overeating

198. Do you think the meal in the photo is healthy? *

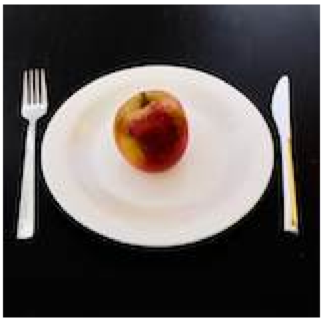

Zaznacz tylko jedną odpowiedź.

- Yes
- No

199. Would you still be hungry immediately after eating this meal? *

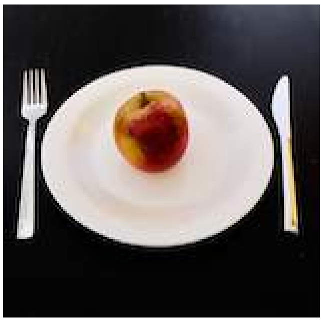

Zaznacz tylko jedną odpowiedź.

- Yes
- No

200. Do you think this meal is for you: *

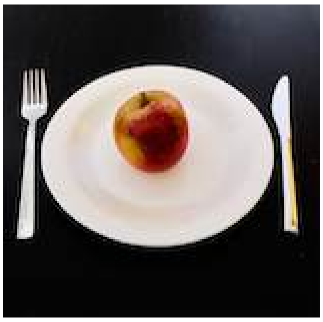

Zaznacz tylko jedną odpowiedź.

- too caloric
- has the right amount of kcal
- it has too few calories, I can or should eat a larger portion

201. How many calories do you think the meal in the photo has? *

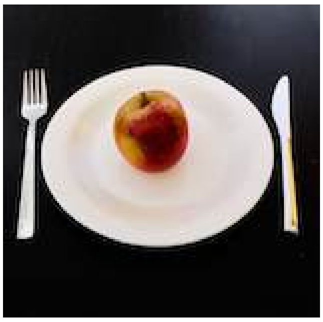

202. How long after eating the meal would you start feeling hungry again? *

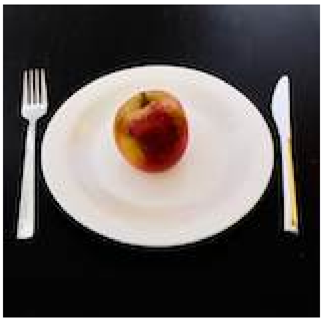

Zaznacz tylko jedną odpowiedź.

- Before the hour is up
- After 1 hour to 2 hours
- Over 2 hours, up to 3 hours
- Over 3 hours

Orange juice without added sugar

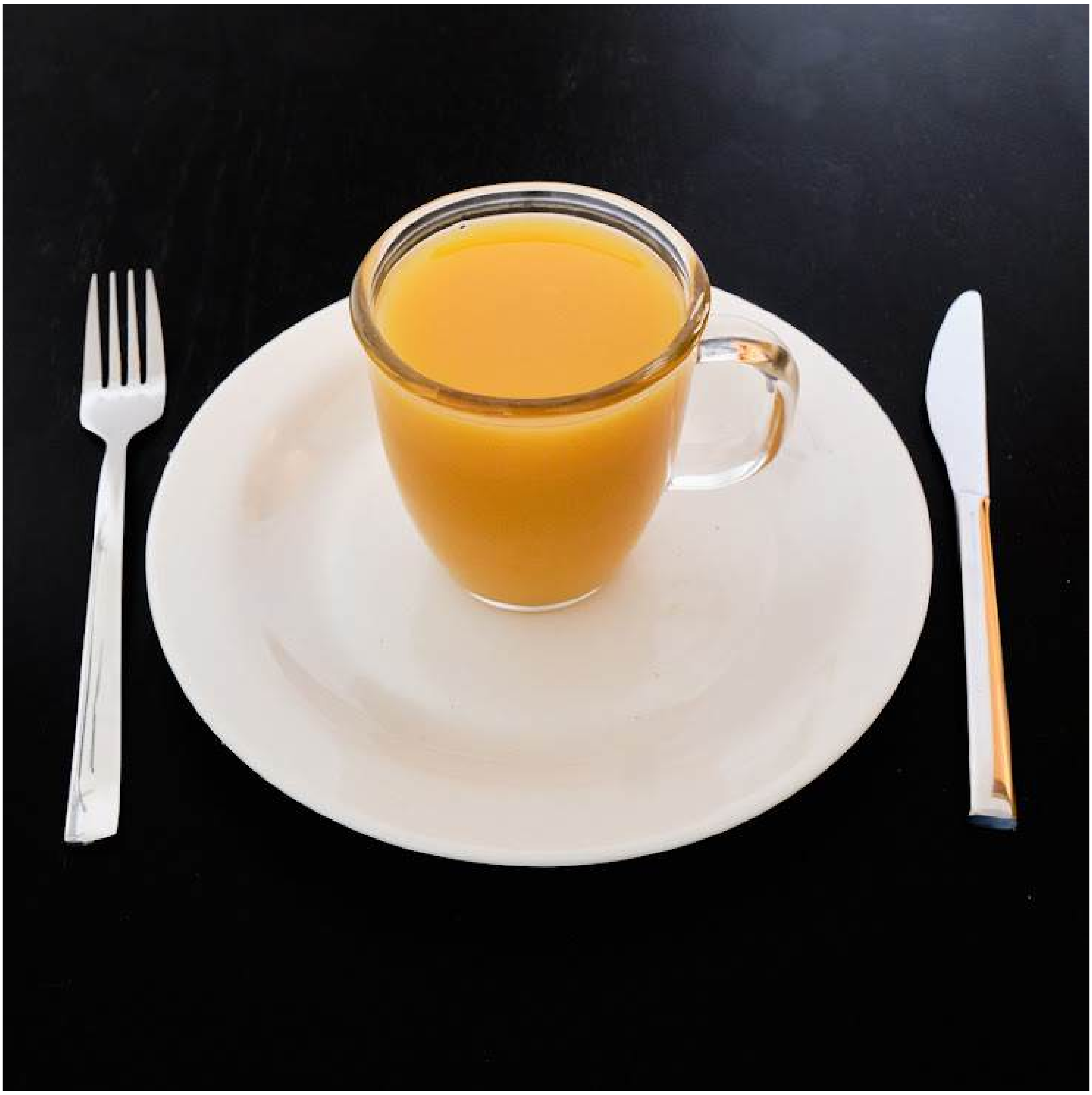

203. Determine the size of the meal shown in the photo *

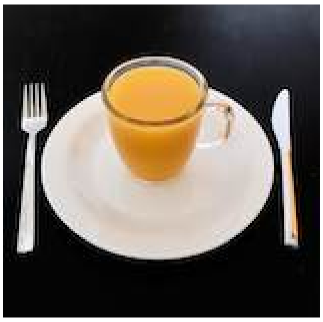

Zaznacz tylko jedną odpowiedź.

Very small, almost nothing

1 ⬭

2 ⬭

3 ⬭

4 ⬭

5 ⬭

6 ⬭

7 ⬭

8 ⬭

9 ⬭

10 ⬭

Very Large

204. How full would you be with this meal? *

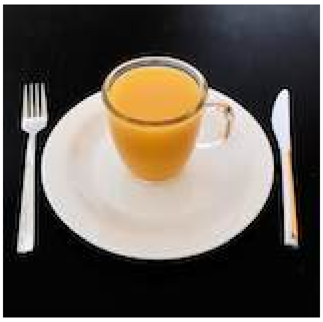

Zaznacz tylko jedną odpowiedź.

	Not full at all, still hungry
1	☐
2	☐
3	☐
4	☐
5	☐
6	☐
7	☐
8	☐
9	☐
10	☐
	Extremely full, on the verge of overeating

205. Do you think the meal in the photo is healthy? *

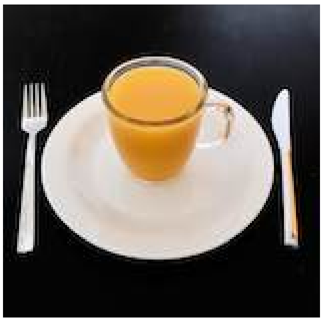

Zaznacz tylko jedną odpowiedź.

- Yes
- No

206. Would you still be hungry immediately after eating this meal? *

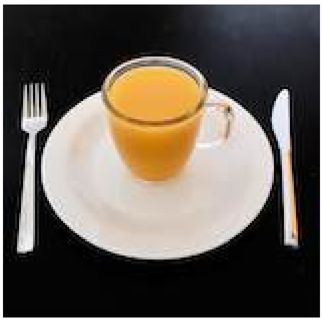

Zaznacz tylko jedną odpowiedź.

- Yes
- No

207. Do you think this meal is for you: *

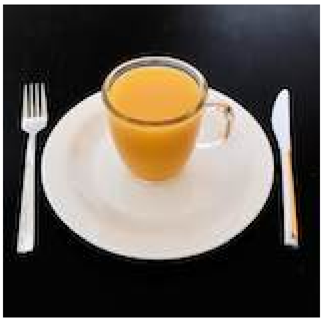

Zaznacz tylko jedną odpowiedź.

- too caloric
- has the right amount of kcal
- it has too few calories, I can or should eat a larger portion

208. How many calories do you think the meal in the photo has? *

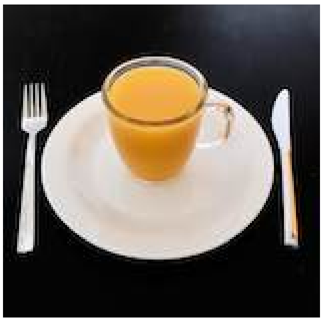

209. How long after eating the meal would you start feeling hungry again? *

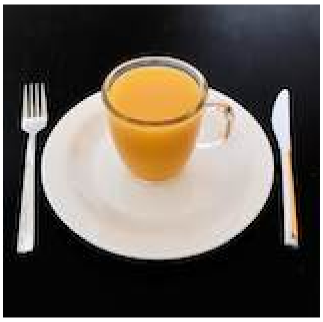

Zaznacz tylko jedną odpowiedź.

- Before the hour is up
- After 1 hour to 2 hours
- Over 2 hours, up to 3 hours
- Over 3 hours

Thank you for completing the survey!

Ta treść nie została utworzona ani zatwierdzona przez Google.

Formularze Google
